# Low Expression of YTH Domain-Containing 1 Promotes Microglial M1 Polarization by Reducing the Stability of Sirtuin 1 mRNA

**DOI:** 10.3389/fncel.2021.774305

**Published:** 2021-12-15

**Authors:** Hongxiu Zhou, Zongren Xu, Xingyun Liao, Shiyun Tang, Na Li, Shengping Hou

**Affiliations:** ^1^The First Affiliated Hospital of Chongqing Medical University, Chongqing, China; ^2^Chongqing Eye Institute, Chongqing, China; ^3^Chongqing Key Laboratory of Ophthalmology, Chongqing, China; ^4^Chongqing Branch of National Clinical Research Center for Ocular Diseases, Chongqing, China; ^5^College of Basic Medicine, Chongqing Medical University, Chongqing, China

**Keywords:** m6A, YTHDC1, microglia cells, SIRT1, RNA stability

## Abstract

The N6-methyladenosine (m6A) modification is the most abundant posttranscriptional mRNA modification in mammalian cells and is dynamically modulated by a series of “writers,” “erasers,” and “readers.” Studies have shown that m6A affects RNA metabolism in terms of RNA processing, nuclear export, translation, and decay. However, the role of the m6A modification in retinal microglial activation remains unclear. Here, we analyzed the single-cell RNA sequencing data of retinal cells from mice with uveitis and found that the m6A-binding protein YTH domain-containing 1 (YTHDC1) was significantly downregulated in retinal microglia in the context of uveitis. Further studies showed that YTHDC1 deficiency resulted in M1 microglial polarization, an increased inflammatory response and the promotion of microglial migration. Mechanistically, YTHDC1 maintained sirtuin 1 (SIRT1) mRNA stability, which reduced signal transducer and activator of transcription 3 (STAT3) phosphorylation, thus inhibiting microglial M1 polarization. Collectively, our data show that YTHDC1 is critical for microglial inflammatory response regulation and can serve as a target for the development of therapeutics for autogenic immune diseases.

## Introduction

Uveitis, which is an autoimmune ophthalmopathy, accounts for approximately 10–15% of legal blindness in Western countries (Nussenblatt, 1990; Wakefield and Chang, 2005; Hou et al., 2015). Furthermore, compared with some common age-related eye diseases, uveitis may have a greater socioeconomic impact because it is more prevalent among younger working-age patients (Suttorp-Schulten and Rothova, 1996; Hou et al., 2020). At present, the etiology of uveitis is still unknown, and the pathogenesis is complicated. However, studies have shown that innate immune cells, including microglia, play a vital role in the induction and exacerbation of uveitis (Rao et al., 2003; Dick et al., 2004; Langmann, 2007; Karlstetter et al., 2015).

Microglia are the resident immune cells of the retina and account for 5–20% of the glial cell population (Ling and Leblond, 1973; Langmann, 2007). In the normal retina, microglia are in a resting state and distributed from the outer plexus layer to the nerve fiber layer, which dynamically expands and retracts to actively monitor the surrounding tissue microenvironment (Chen et al., 2002; Nimmerjahn et al., 2005; Rathnasamy et al., 2019). Subtle changes in the retinal microenvironment can trigger robust microglial activation. Activated microglia migrate to the subretinal space, recruit peripheral inflammatory cells into the retina, and then exacerbate retinal damage, which initiates many retinal diseases (Gupta et al., 2003; Madeira et al., 2015; Rathnasamy et al., 2019). Microglia can be activated by LPS or signals associated with infection, such as IFN-γ, into a proinflammatory phenotype (M1) *in vitro* and then produce proinflammatory cytokines and oxidative metabolites and present antigens (Orihuela et al., 2016; Wang et al., 2016). Cytochrome c oxidase subunit II (COX2), inducible nitric oxide synthase (iNOS), CD16, CD86 and major histocompatibility complex II (MHC-II) are phenotypic markers of M1 microglia (Chhor et al., 2013; Lan et al., 2017). M1 microglia respond to injury or infection and serve as the first line of defense in the innate immune system, but an excessive neuroinflammatory response tends to exacerbate neuronal death and promote tissue inflammation and damage (Block et al., 2007). Therefore, exploring the underlying mechanism of microglial activation is of great importance for understanding the pathological process of disease and discovering the potential regulators of innate immunity.

In recent years, epigenetic modifications of RNA have attracted increasing attention, especially the m6A modification, which is the most abundant form of posttranscriptional mRNA modification in eukaryotic cells (Dubin and Taylor, 1975; Dominissini et al., 2012; Roundtree et al., 2017a). It has been shown that the m6A modification dominates many cellular and biological processes and participates in the pathological processes of various diseases (Geula et al., 2015; Lin et al., 2016; Zhang et al., 2017; Tan and Gao, 2018). The m6A modification affects RNA metabolism through RNA processing, nuclear export, RNA translation and decay, and these outcomes are mediated by m6A readers, writers and erasers (Zhou et al., 2015; Haussmann et al., 2016; Roundtree et al., 2017b; Zhang et al., 2020). YTHDC1 is an m6A modification reader that is primarily localized in the nucleus but can shuttle into the cytoplasm and preferentially recognize GG (m6A) C sequences (Xu et al., 2014). Previous studies have shown that YTHDC1 participates in the variable splicing of precursor mRNA through interactions with splicing factors (Adhikari et al., 2016; Xiao et al., 2016) and plays a role in mRNA nuclear export (Roundtree et al., 2017b) and RNA stabilization (Shima et al., 2017). However, it is still unclear whether YTHDC1 is involved in the regulation of microglial inflammation.

In the present study, we first showed that the expression of the m6A reader YTHDC1 was downregulated in retinal microglia in two animal models of uveitis. Knockdown of YTHDC1 resulted in increased expression of M1 phenotypic markers and proinflammatory cytokines in BV2 cells and promoted microglial migration. Mechanistically, YTHDC1 restrained M1 microglial activation by decreasing STAT3 phosphorylation. Furthermore, YTHDC1 maintained SIRT1 mRNA stability to reduce STAT3 acetylation, which in turn reduced STAT3 phosphorylation. In conclusion, our study elucidates the pivotal role of YTHDC1 in M1 microglial activation and suggests that the m6A modification may participate in the regulation of uveitis.

## Materials and Methods

### Cell Culture

The BV2 cell line was generously provided by Professor Zhifang Dong of the Children’s Hospital of Chongqing Medical University. Dulbecco’s modified Eagle’s medium (DMEM)/F12 (Gibco, NY, United States) containing 10% fetal bovine serum (FBS) (Gibco, NY, United States) and 1% penicillin/streptomycin (Beyotime, Shanghai, China) was used to culture BV2 cells. When the cells reached approximately 80% confluence, they were harvested or subcultured at a ratio of 1:4.

### Cell Stimulation

According to the experimental requirements, BV2 cells were seeded in plates at different densities, such as 1 × 10^6^ cells/well for RT-qPCR and 3 × 10^4^ cells/well for immunofluorescence assays. The next day, the cells were serum-starved for 12 h, and then 1 μg/ml LPS (Sigma-Aldrich, St. Louis, MO, United States) was added to the culture medium for an additional 24 h. LPS concentrations were selected based on previous study (Liao et al., 2020). Cells cultured in medium without LPS were used as controls.

In the SIRT1 activation experiments, the cells were treated with SRT1720 (Selleck, Houston, TX, United States; 10 μM) for 24 h before LPS was added. Cells that were not treated with LPS or SRT1720 were used as blank controls.

### Lentiviral Transfection

To knockdown the YTHDC1 gene (NM_177680), shRNA sequences were designed by Shanghai GeneChem Co., Ltd (China). The shRNA sequences for murine YTHDC1 and the negative control sequence were cloned into hU6-MCS-CBh-gcGFP-IRES-puromycin to generate the GV493 lentiviral vectors. The lentivirus was transfected into BV2 cells based on the manufacturer’s instructions. In short, BV2 cells were seeded and transfected with the negative control lentivirus or the YTHDC1-specific shRNA lentivirus at an MOI of 80 for 10 h. The efficiency of lentivirus infection was determined by calculating the GFP percentage by fluorescence microscopy after transfection for 72 h. Then, 3 μg/ml puromycin was added to the medium to remove the untransfected cells. RT-qPCR and Western blotting were used to determine the efficiency of shRNA knockdown. All shRNA sequences and negative control sequences are shown in Table 1.

**TABLE 1:** All shRNA sequences and negative control sequences.

Name	Sequences
Ythdc1-RNAi (ShYTHDC1-1)	tgAGGTCGTTATCGAAGATAA
Ythdc1-RNAi (ShYTHDC1-2)	tgATATGAGGGTCGATGATTT
Ythdc1-RNAi (ShYTHDC1-3)	tgGATTTGCAGGCGTGAATTA
Negative Control (NC)	tgTTCTCCGAACGTGTCACGT

### RNA Extraction and Quantitative Reverse Transcription PCR

TRIzol reagent (Roche, CA, United States) was used to extract and purify total RNA from cells according to the manufacturer’s instructions and previous study (Zhao et al., 2020). Cytoplasmic and nuclear RNA were isolated according to the specifications of the Cytoplasmic & Nuclear RNA Purification Kit (Norgen, Canada). cDNA was reverse-transcribed from equal amounts of RNA by the PrimeScript™ RT reagent kit (Takara, Kyoto, Japan). The levels of RNA transcripts were measured by SYBR Green (Bio-Rad, CA, United States) on an Applied Biosystems 7500 PCR system (United States). The housekeeping gene β-actin was used as the reference gene. All the primers are listed in Supplementary Table 1.

### Western Blotting

The western blot protocol can be found in our previous study (Huang et al., 2018). RIPA lysis buffer (Beyotime, Shanghai, China) containing 1 mM PMSF (Beyotime, Shanghai, China) was used to extract total proteins from cells. The cell lysate was collected and placed on ice for 30 min. The cell lysate was then centrifuged at 14,000 × *g* for 15 min at 4 °C, and then the supernatants were collected. A BCA colorimetric protein assay kit (Beyotime, Shanghai, China) was used to measure the protein concentrations. Equal amounts of protein (15 μg) were separated by 8–15% SDS-PAGE (Boster Biological Technology, Wuhan, China) and transferred to a PVDF membrane (Millipore, Burlington, MA, United States). The membranes were blocked in Tris-buffered saline plus Tween-20 (TBST) containing 5% fat-free milk for 2 h at room temperature. Subsequently, the membranes were incubated in proportionally diluted specific primary antibodies overnight at 4°C. After being washed with TBST three times, the membranes were incubated with secondary antibodies for 1 h at room temperature and visualized by an imaging system (VILBER, Germany). The relative protein expression was normalized to GAPDH. All antibodies used are shown in Supplementary Table 2.

### Immunocytochemistry

Cells were seeded on slides, and then the cells were treated accordingly based on the experimental groupings. The next day, the slides were soaked in 5% goat serum containing 0.3% Triton X-100 for 30 min at room temperature. Then, rabbit anti-iNOS (1:200, Abcam, Cambridge, United Kingdom) and goat anti-Iba1 (1:500, Abcam, Cambridge, United Kingdom) primary antibodies were added to the slides and incubated overnight at 4°C. After being washed with phosphate-buffered saline (PBS) three times, the slides were incubated in Alexa Fluor 488-labeled goat anti-rabbit IgG and Cy3-labeled donkey anti-goat IgG secondary antibodies (1:500, Beyotime, Shanghai, China) for 1 h at room temperature, and DAPI was used to stain the cell nuclei for 5 min. Details can be found in our previous study (Huang et al., 2018). The slides were observed under a microscope (Leica, Germany) after being washed with PBS buffer.

### Transwell Migration Assay

A 24-well plate with an 8 μm pore size Transwell filter (Corning, NY, United States) was used to perform the migration assay. The upper chamber was seeded with 3 × 10^4^ cells cultured in 200 μl of serum-free medium, and 500 μl of medium containing 10% FBS was added to the bottom chamber. The cells were cultured for 6 h, and the cells on the upper side of the Transwell filter were removed. Crystal violet (Solarbio, Beijing, China) was used to stain the cells on the underside of the membrane after immobilization by paraformaldehyde. Details can be found in previous study (Yu et al., 2020). Then, four regions were randomly selected, the number of migrated cells was counted under a microscope.

### Wound Healing Assay

BV2 cells were seeded in 24-well plates at a density of 3 × 10^5^ cells/well and incubated for 24 h. Subsequently, an area was removed by scratching the cell monolayer with a plastic pipette tip perpendicular to the plate. PBS was used to wash away the detached cells, and the medium was changed to serum-free medium. Photomicrographs of identical areas in each well were taken at 0 and 6 h. Details can be found in previous study (El Gaamouch et al., 2020). Change in the scratch width were analyzed by ImageJ software.

### Total mRNA N6-Methyladenosine Quantification

The level of m6A in total mRNA was measured based on the specifications of the EpiQuik™ m6A RNA Methylation Quantification Kit (Epigentek, United States). This assay is widely used, as demonstrated by previous studies (Zhang et al., 2016). Briefly, 200 ng of RNA was added to each well, capture and detection antibodies were subsequently mixed in wells. After being incubated, the plates were washed several times, the absorbance of each well was measured at 450 nm, and the level of m6A was calculated according to the following formula: $\mathrm{m6A}\%=\frac{\left(\mathrm{sample}\mathrm{OD}-\mathrm{NC}\mathrm{OD}\right)÷S}{\left(\mathrm{PC}\mathrm{OD}-\mathrm{NC}\mathrm{OD}\right)÷P}\times 100\%$. NC is the negative control, and PC is the positive control. S is the RNA sample input quantity, and P is the positive control input quantity. In this experiment, S = 200 ng and P = 1 ng.

### mRNA Stability Assay

The cells were treated based on the experimental groupings, and 5 μg/ml actinomycin D (ActD; Sigma) was added to the medium to inhibit global mRNA transcription. The cells were harvested at specific points, RNA was isolated, and the RNA of the genes of interest were quantified by RT-qPCR. mRNA stability was determined by analyzing the relative expression at 0 h after actinomycin D treatment. The protocol can be found according to the previous study (Roundtree et al., 2017b).

### Methylated RNA Immunoprecipitation qPCR

Total RNA was extracted as described previously, and MeRIP was performed with an MeRIP kit (BersinBio, Guangzhou, China) in accordance with the manufacturer’s recommendations. In short, the RNA was randomly fragmented into 100-nucleotide fragments, and anti-m6A antibodies or anti-rabbit IgG linked with A/G magnetic beads was used for immunoprecipitation. A magnetic frame was used to elute the m6A-precipitated RNA. Phenol-chloroform and ethanol precipitation were used to extract the enriched RNA (Wang et al., 2019).

Potential m6A modification sites on SIRT1 mRNA were predicted in RMBase v2.0 (Xuan et al., 2018) and SRAMP (Zhou et al., 2016). The two most likely sites (SIRT1-1 and SIRT1-2) were selected, and primers for qPCR analysis were designed. All primers for MeRIP-qPCR are listed in Supplementary Table 3. The quantitative expression of m6A enrichment is expressed as the enrichment percentage relative to the input sample (% input) and was calculated as follows: % Input = 2^Ct(Input)–Ct(MeRIP)^ × (input dilution factor) × 100%. The input dilution factor is the proportion of input RNA in m6A-IP RNA. For example, if a 100 μl sample was used for MeRIP and a 10 μl sample was used as input, the value of the input dilution factor was 1/10.

### Statistics

Statistical analysis was performed using SPSS 26.0 software, and GraphPad Prism 8.0 software was used for mapping. All data are presented as the mean ± SD. For statistical comparisons, we first evaluated whether the data were normally distributed and then evaluated the homogeneity of variance between normally distributed data. Data with a normal distribution and homogeneous variance were compared by Student’s *t*-test. If *P* < 0.05, the difference was statistically significant.

## Results

### YTH Domain-Containing 1 Expression in Microglia Negatively Correlated With Inflammation

**FIGURE 1:**
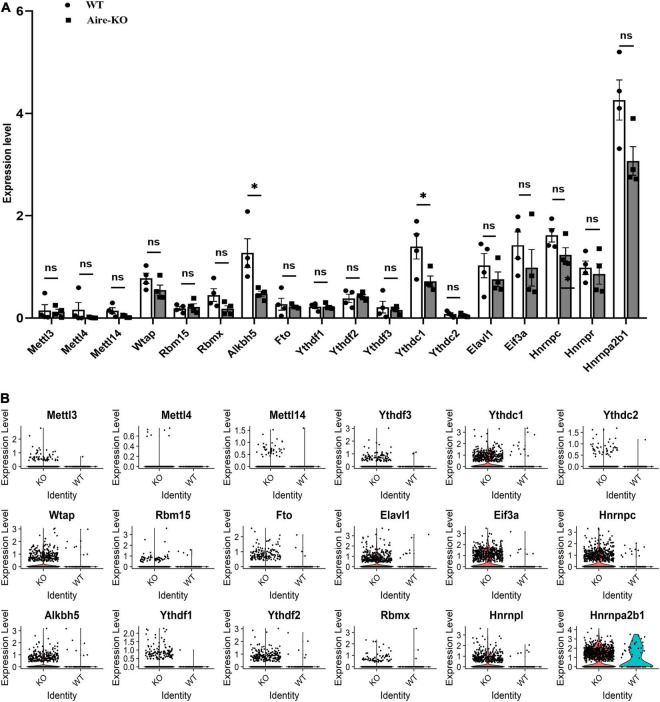
scRNA-seq analysis of Aire^– /–^ retinal microglia. **(A)** Bar charts showing the expression levels of m6A writers, erasers and readers in WT and Aire^–/–^ retina microglia (*n* = 4). **(B)** Violin plots showing the expression levels of m6A writers, erasers and readers in WT and Aire^–/–^ retinal microglia. *P < 0.05 by Student’s *t*-test; n.s., no significance. The data are presented as the mean ± SD.

**FIGURE 2:**
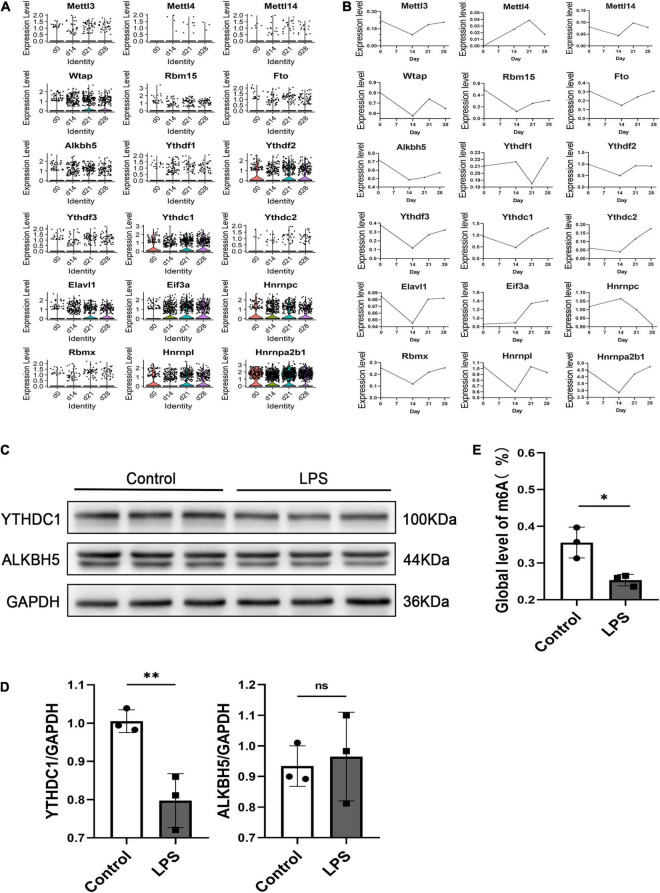
scRNA-seq analysis of EAU mouse retinal microglia and ALKBH5 and YTHDC1 protein expression levels in M1 microglia. **(A)** Violin plots showing the expression level of m6A genes in retinal microglia at 0, 14, 21, and 28 days. **(B)** Line plots showing the expression levels of m6A genes in retinal microglia at 0, 14, 21, and 28 days. **(C,D)** Western blot analysis of ALKBH5 and YTHDC1 in LPS (1 μg/ml)-induced BV2 cells after 24 h compared with those of the control (*n* = 3). **(E)** Quantitative m6A RNA methylation analysis (colorimetric) of LPS (1 μg/ml)-induced BV2 cells at 24 h compared with that of the control (*n* = 3). *P < 0.05 and ^**^P < 0.01 by Student’s *t*-test; n.s., no significance. The data are presented as the mean ± SD.

To investigate the role of the m6A modification in retinal microglia in the context of uveitis, we first examined the mRNA levels of m6A writers, erasers and readers in microglia using GEO data (GSM3854512-3854519) from single-cell RNA-seq analysis of an Aire^–/–^ spontaneous uveoretinitis mouse model (Heng et al., 2019). The results showed conspicuously reduced alkB homolog 5 (ALKBH5) and YTHDC1 expression in microglia in Aire^–/–^ retinas (*P* < 0.05) (Figures 1A,B). Moreover, we analyzed the single-cell RNA-seq data from experimental autoimmune uveitis (EAU) retinas of our other study (unpublished observations). We found that ALKBH5 and YTHDC1 in microglia were also downregulated on the 14th day, which corresponded to peak retinal inflammation, and upregulated on the 21st and 28th days, which represented the recovery periods (Figures 2A,B). To confirm the changes in the protein levels of ALKBH5 and YTHDC1 in microglia, we used LPS to induce the M1 phenotype in BV2 cells *in vitro*, which increased the expression of iNOS (*P* < 0.01), COX2 (*P* < 0.05) and tumor necrosis factor-α (TNF-α) (*P* < 0.01) (Supplementary Figures 1A–C) and promoted the migration of BV2 cells (*P* < 0.05 and *P* < 0.001) (Supplementary Figures 1D–G). The results revealed that only the YTHDC1 protein level was markedly downregulated in M1 microglia (*P* < 0.01) (Figures 2C,D). In addition, we quantitatively measured the global level of m6A in the total RNA of BV2 cells. The m6A level was decreased in LPS-treated BV2 cells (*P* < 0.05) (Figure 2E). Taken together, these results suggested that the m6A modification in retinal microglia in the context uveitis was changed during the inflammatory stage and that YTHDC1 was expressed at low levels in inflammatory microglia.

### YTH Domain-Containing 1 Inhibited M1 Microglial Activation

**FIGURE 3:**
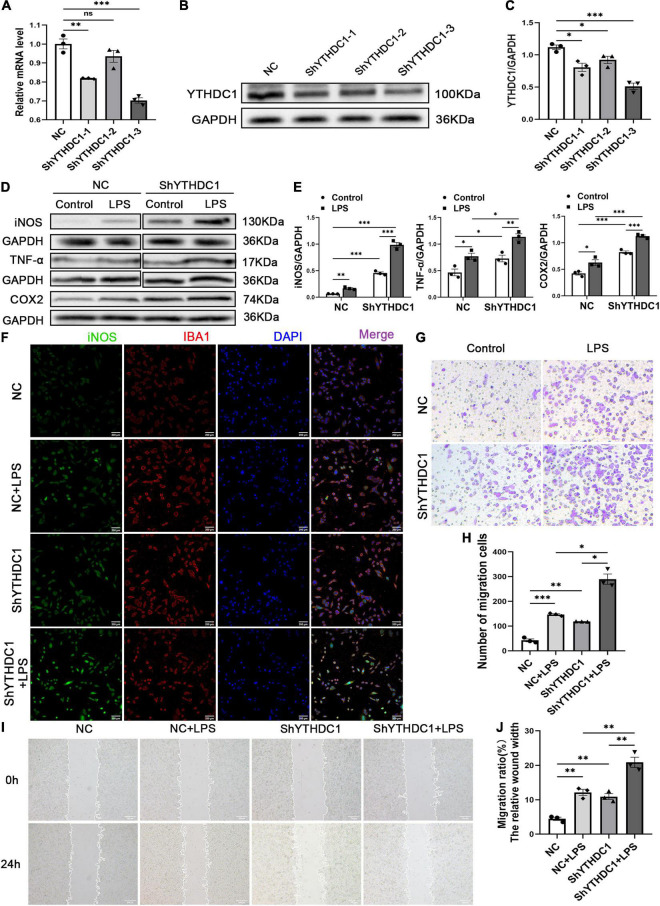
YTHDC1 depletion facilitates BV2 microglia M1 activation and migration *in vitro*. **(A–C)** The transfection efficiency of YTHDC1 knockdown in BV2 cells was measured by qRT-PCR **(A)** and Western blotting **(B,C)**. **(D,E)** Western blot analysis of iNOS, COX2 and TNF-α in the different groups. **(F)** Immunofluorescence analysis of iNOS in the different groups. **(G,H)** Wound healing assays in the different groups. **(I,J)** Transwell assays in the different groups. NC: cells transfected with negative control shRNA; ShYTHDC1 (1, 2, and 3) or ShYTHDC1: cells transfected with YTHDC1 shRNA. *P < 0.05, ^**^P < 0.01, and ^***^P < 0.001 by Student’s *t*-test. The data are presented as the mean ± SD. (*n* = 3). Scale bar, 200 μm.

To further explore the regulatory effect of YTHDC1 on microglial cells, we used ShYTHDC1 (1, 2, and 3) and ShNC lentiviruses to knock down YTHDC1 expression in BV2 cells. After treatment with puromycin, approximately 90% of all lentiviruses infected BV2 cells were GFP^+^ cells (Supplementary Figure 1H). The RNA and protein expression levels of YTHDC1 were lower in BV2 cells transfected with ShYTHDC1 (1, 2, and 3) than in those transfected with ShNC (*P* < 0.05, *P* < 0.01, and *P* < 0.001, respectively) (Figures 3A–C). ShYTHDC1-3 was used in the subsequent experiments because it had the highest knockdown efficiency. Western blot analysis indicated that YTHDC1 deficiency facilitated the expression of the M1 microglial markers iNOS and COX2 and the inflammatory cytokine TNF-α (*P* < 0.05, *P* < 0.01, and *P* < 0.001, respectively) (Figures 3D,E). In addition, the immunofluorescence assay showed that iNOS expression was also increased after YTHDC1 silencing (Figure 3F). Cell migration was substantially promoted by silencing YTHDC1 (*P* < 0.05, *P* < 0.01, and *P* < 0.001, respectively) (Figures 3G–J). In summary, we believe that YTHDC1 deficiency accelerate microglial M1 activation and migration.

### Knocking Down YTH Domain-Containing 1 Reduced Sirtuin 1 Expression in Microglia

**FIGURE 4:**
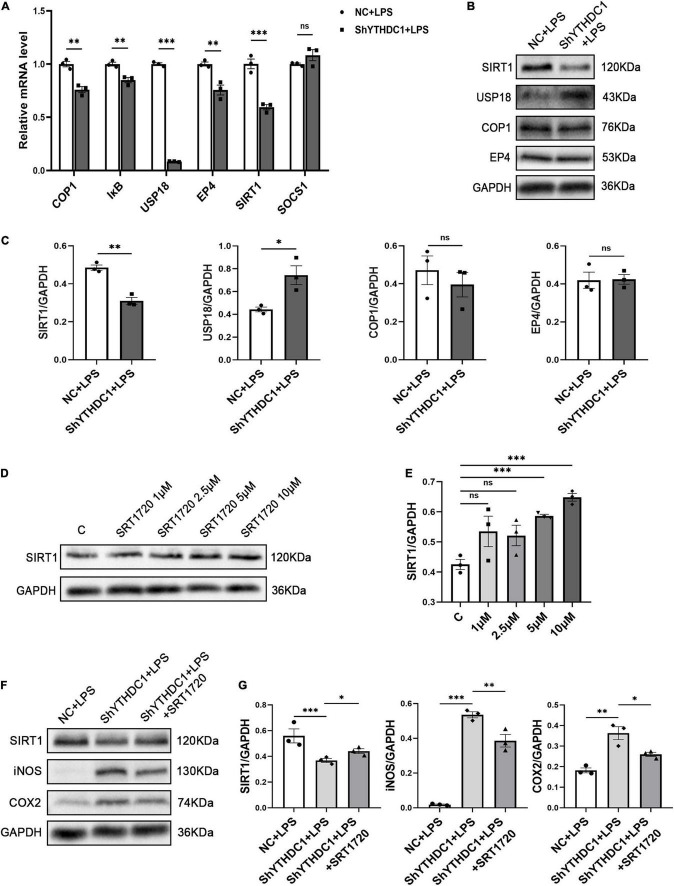
SIRT1 is the target of YTHDC1 in BV2 cells. **(A)** RT-qPCR analysis of COP1, IκB, USP18, EP4, SIRT1, and SOCS1 in ShYTHDC1-transfected BV2 cells compared to unloaded lentivirus-transfected BV2 cells after treatment with LPS. **(B,C)** Western blot analysis of COP1, USP18, EP4, and SIRT1 in ShYTHDC1-transfected BV2 cells compared to unloaded lentivirus-transfected BV2 cells after treatment with LPS. **(D,E)** Western blot analysis of SIRT1 protein expression in BV2 cells treated with different concentrations of SRT1720 compared with the control group. **(F,G)** Western blot analysis of SIRT1, iNOS and COX2 in the different groups. *P < 0.05, ^**^P < 0.01, and ^***^P < 0.001 by Student’s *t*-test. The data are presented as the mean ± SD (*n* = 3).

To understand the underlying mechanism by which YTHDC1 exerts effects on microglial polarization, we considered the impact of YTHDC1 on RNA nuclear export and RNA stability and hypothesized that YTHDC1 regulates some anti-inflammatory genes in microglia. We used qRT-PCR to validate genes that were shown in the literature to inhibit microglial inflammation (Woodling et al., 2014; Goldmann et al., 2015; Chen et al., 2018; Subedi et al., 2018; Zhang et al., 2019; Ndoja et al., 2020). The results revealed that the expression of E3 ubiquitin ligase (COP1), IκB, ubiquitin specific peptidase 18 (USP18), prostaglandin E receptor 4 (EP4) and SIRT1 was decreased by YTHDC1 knockdown after treatment with LPS (*P* < 0.01 and *P* < 0.001) (Figure 4A). Then, COP1, USP18, EP4 and SIRT1, which exhibited obvious changes were selected, and their protein levels were measured. The results showed that YTHDC1 deficiency reduced the protein level of SIRT1 (*P* < 0.05 and *P* < 0.01) (Figures 4B,C). Next, we used the activator SRT1720 to increase the activity of SIRT1 (Feige et al., 2008). And we found that SRT1720 at the concentration of 5 μm or 10 μm increased the SIRT1 expression in BV2 cells (*P* < 0.001 and *P* < 0.001) (Figures 4D,E). Finally, we pretreated YTHDC1-knockdown BV2 cells with 10 μm SRT1720, which effectively reduced the LPS-induced high expression of iNOS and COX2 (*P* < 0.01 and *P* < 0.05) (Figures 4F,G).

### Sirtuin 1 Regulated Signal Transducer and Activator of Transcription 3 Phosphorylation Through Deacetylation in Microglia

**FIGURE 5:**
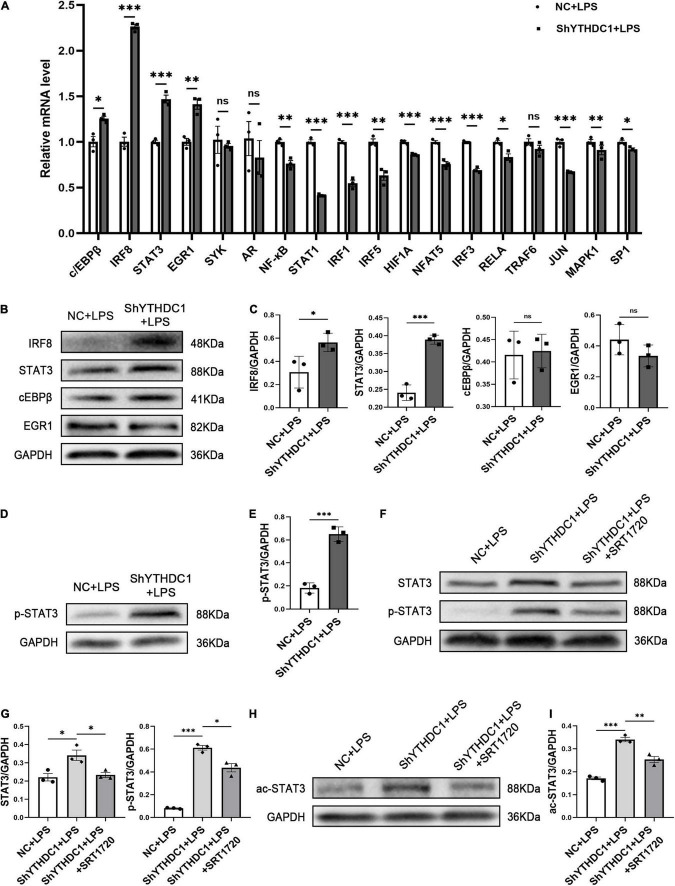
STAT3 phosphorylation is regulated by SIRT1 in BV2 after YTHDC1 silencing. **(A)** RT-qPCR analysis of some proinflammatory transcription factors in YTHDC1-knockdown BV2 cells after treatment with LPS. **(B,C)** Western blot analysis of the protein levels of IRF8, STAT3, cEBPβ, and EGR1 in YTHDC1-knockdown BV2 cells after treatment with LPS. **(D,E)** Western blot analysis of p-STAT3 in NC- or shYTHDC1-transfected BV2 cells after treatment with LPS. **(F,G)** Western blot analysis of STAT3 and p-STAT3 in the different groups. **(H,I)** Western blot analysis of ac-STAT3 in the different groups. *P < 0.05, ^**^P < 0.01, and ^***^P < 0.001 by Student’s *t*-test. The data presented as the mean ± SD (*n* = 3).

Given that YTHDC1 knockdown reduced SIRT1 expression in microglia, we further investigated how this change promoted microglial M1 polarization. The activation of proinflammatory transcription factors is required for microglial M1 polarization, and we examined the expression of some proinflammatory transcription factors by qRT-PCR. The results showed that YTHDC1 depletion significantly upregulated c/EBPβ, interferon regulatory factor 8 (IRF8) (*P* < 0.001), STAT3 (*P* < 0.001) and early growth response 1 (EGR1) (*P* < 0.01) mRNA levels (Figure 5A). However, subsequent Western blot analysis showed that only IRF8 (*P* < 0.05) and STAT3 (*P* < 0.001) protein levels were elevated (Figures 5B,C). Furthermore, the Western blot results also showed that STAT3 phosphorylation levels were increased by YTHDC1 knockdown (*P* < 0.001) (Figures 5D,E).

To confirm the regulatory effect of SIRT1 on STAT3 in microglia, we pretreated YTHDC1-knockdown BV2 cells with SRT1720 and then evaluated the expression of STAT3 and pSTAT3. As expected, the upregulated expression levels of STAT3 and pSTAT3 in ShYTHDC1-treated cells were downregulated after treatment with the SIRT1 activator (*P* < 0.05 and *P* < 0.05) (Figures 5F,G). SIRT1 is a nicotinamide adenine dinucleotide (NAD+)-dependent enzyme with deacetylation activity, and it has been reported that STAT3 acetylation may be an important condition to promote its phosphorylation and enhance its dimerization in the nucleus (Ray et al., 2008; Nie et al., 2009). Therefore, we measured the acetylation level of STAT3 with or without SRT1720 in YTHDC1-depleted BV2 cells. SRT1720 effectively reduced the upregulated STAT3 acetylation level in ShYTHDC1-treated cells (*P* < 0.01) (Figures 5H,I).

Collectively, these data suggested that YTHDC1 deficiency in microglia promoted STAT3 acetylation by reducing SIRT1 expression, which then increased the phosphorylation level of STAT3, thus accelerating microglial M1 activation.

### YTH Domain-Containing 1 Silencing Reduced the Stability of Sirtuin 1 mRNA in Microglia

**FIGURE 6:**
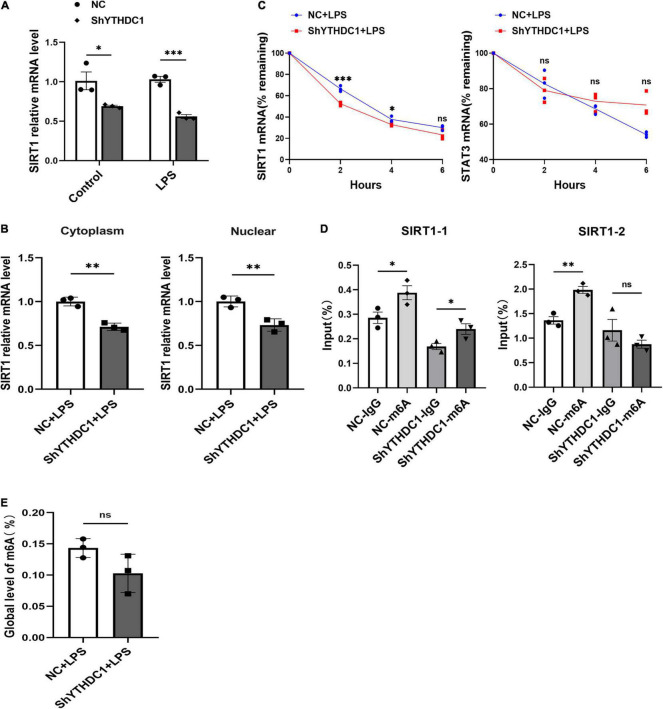
YTHDC1 promoted the stability of SIRT1 mRNA in BV2 cells. **(A)** RT-qPCR analysis of SIRT1 in NC- or shYTHDC1-transfected BV2 cells after treatment with LPS. β-actin served as a loading control. **(B)** RT-qPCR analysis of SIRT1 mRNA levels in the cytoplasm and nucleus of NC- or shYTHDC1-transfected BV2 cells after treatment with LPS. **(C)** RT-qPCR analysis of SIRT1 and STAT3 mRNA levels in NC- or shYTHDC1-transfected BV2 cells subjected to ActD (1 μg/ml) treatment for the indicated times after treatment with LPS. **(D)** MeRIP analysis followed by qRT-PCR was used to assess the m6A modification of SIRT1 in NC- or shYTHDC1-transfected BV2 cells. **(E)** Quantitative detection of m6A RNA methylation in NC- or shYTHDC1-transfected BV2 cells after treatment with LPS. *P < 0.05, ^**^P < 0.01, and ^***^P < 0.001 by Student’s *t*-test. The data are presented as the mean ± SD (*n* = 3).

We found that YTHDC1 affected the expression of SIRT1 mRNA in microglia with or without LPS induction (*P* < 0.05 and *P* < 0.001) (Figures 4A, 6A). As a nuclear reader of m6A modification, the most notable role of YTHDC1 is to affect mRNA nuclear export. Therefore, YTHDC1 silencing may decrease SIRT1 transcription and translation via inhibiting its mRNA nuclear export. We examined the subcellular distribution of SIRT1 mRNA in microglia after YTHDC1 silencing. Unfortunately, the mRNA levels of SIRT1 were downregulated in both the cytoplasm and nucleus (*P* < 0.01 and *P* < 0.01) (Figure 6B). Since YTHDC1 may regulate RNA stability (Shima et al., 2017), we measured the half-life of SIRT1 and STAT3 mRNA in YTHDC1-depleted BV2 cells. YTHDC1 silencing significantly decreased the stability of SIRT1 mRNA but did not affect the stability of STAT3 mRNA (Figure 6C). In addition, the MeRIP-qPCR assay showed that the m6A modification was enriched in SIRT1 mRNA (*P* < 0.05 and *P* < 0.01) (Figure 6D), YTHDC1 may regulate SIRT1 expression by recognizing these sites. However, there was no significant change in the global level of m6A after silencing YTHDC1 in microglia (*P* > 0.05) (Figure 6E).

## Discussion

Although research on RNA methylation, a type of posttranscriptional epigenetic modification, began in the 1970s, due to technical limitations, it was only recently that continuous breakthroughs in understanding the m6A modification have occurred (Jia et al., 2011; Zhao et al., 2017; Zheng et al., 2020). A growing body of evidence suggests that m6A profoundly contributes to multiple cellular processes (Geula et al., 2015; Zhang et al., 2017; Wang et al., 2020). However, the effect of the m6A modification on microglia is still largely unknown. We used high-throughput sequencing data to analyze the expression of m6A methyltransferases, demethylases and readers in retinal microglia in uveitis and found that YTHDC1 was expressed at significantly lower levels in microglia undergoing uveitis than in normal. Knockdown of YTHDC1 induced M1 microglial polarization, exacerbated inflammation, and promoted the migration of BV2 cells *in vitro*. Mechanistically, we found that SIRT1 was modulated by YTHDC1 through changes in SIRT1 mRNA stability in microglia.

YTH domain-containing 1, which is mainly distributed in the nucleus, is an important reader of the m6A modification that widely regulates eukaryotic transcripts (Xu et al., 2014). Evidence has indicated that YTHDC1 is associated with the occurrence and development of some tumors (Luxton et al., 2019; Liu et al., 2020). However, the role of YTHDC1 in cellular immune regulation is still unclear. In the present study, we first identified that the decrease in YTHDC1 expression was accompanied by a more severe proinflammatory response in microglia. Using stable YTHDC1-knockdown cells, we then showed that YTHDC1 restrained M1 polarization and migration in BV2 cells *in vitro*. To further clarify the role of YTHDC1, SIRT1 was shown to be the downstream target of YTHDC1 by screening.

Sirtuin 1 is a deacetylase that is involved in many physiological and pathological processes, such as apoptosis, senescence and regulating the inflammatory response (Melhem et al., 2016; Imperatore et al., 2017). A previous study showed that increasing SIRT1 activity promoted the transformation of M1 microglia to the M2 phenotype and reduced the neuroinflammatory response after traumatic brain injury (Chen et al., 2018). In addition, the AMPK-SIRT1 axis inhibits nuclear factor κB (NF-κB) activation by mediating the deacetylation of downstream pathway factors, reducing neuroinflammation in mice with experimental autoimmune encephalomyelitis (EAE) (Parodi et al., 2015). Similar conclusions were found in our study: YTHDC1 silencing downregulated the expression of SIRT1, resulting in increased downstream STAT3 acetylation level, and that caused the promotion of STAT3 phosphorylation. These effects induce M1 microglial activation and exacerbate the inflammatory response.

As a nuclear reader, one of the most dramatic effects of YTHDC1 is RNA nuclear export. YTHDC1 mediates mRNA nuclear export by interacting with the splicing factor and nuclear export adaptor protein SRSF3 (Roundtree et al., 2017b). However, our findings did not indicate that YTHDC1 affected the nuclear export of SIRT1 mRNA. In addition, YTHDC1 interacts with splicing factors to participate in the variable splicing of precursor mRNAs (Xiao et al., 2016). A recent study showed that in mouse embryonic stem cells, YTHDC1 regulates the formation of H3K9me3 and heterochromatin, thus silencing retrotransposon elements and restricting the transformation of these cells to 2C-like cells (Liu et al., 2021). Previous studies have reported that YTHDC1 could regulate RNA stability (Shima et al., 2017). In the present study, we found that SIRT1 mRNA stability was regulated by YTHDC1, as shown by an mRNA stability assay. In addition, a MeRIP-qPCR assay revealed the m6A enrichment in SIRT1 mRNA at two predicted sites. This enrichment may bring about decreased SIRT1 protein levels in YTHDC1-silenced BV2 cells. To the best of our knowledge, this is the first report that YTHDC1 regulates SIRT1 expression through m6A modifications. Our results link YTHDC1-induced microglial activation with acetylation.

Although we reported for the first time that YTHDC1 regulated microglial activation by impacting SIRT1 mRNA stability, there are still some limitations in our study. When looking for downstream targets of YTHDC1, we used RT-qPCR and Western blotting to examine some genes of interest rather than screening target genes based on high-throughput MeRIP-seq. Therefore, the present study might have omitted some m6A-associated candidates that are specifically involved in microglial activation, which deserves further investigation. Additionally, whether YTHDC1 has the same effect on retinal microglia in vivo requires further study. Finally, the possibility that YTHDC1 may function in other roles in uveitis progression should be explored.

## Data Availability Statement

The original contributions presented in the study are included in the article/Supplementary Material, further inquiries can be directed to the corresponding author/s.

## Author Contributions

HZ and NL performed the literature review, planned the experiments, and performed data interpretation. HZ performed most of the experiments. ZX performed the single-cell RNA-seq analysis. XL and ST were involved in optimization of the experimental protocols. SH and NL supervised the study and performed data interpretation. All authors contributed to writing and editing the manuscript.

## Conflict of Interest

The authors declare that the research was conducted in the absence of any commercial or financial relationships that could be construed as a potential conflict of interest.

## Publisher’s Note

All claims expressed in this article are solely those of the authors and do not necessarily represent those of their affiliated organizations, or those of the publisher, the editors and the reviewers. Any product that may be evaluated in this article, or claim that may be made by its manufacturer, is not guaranteed or endorsed by the publisher.

## Abbreviations

m6A: N6-methyladenosine
YTHDC1: YTH domain-containing 1
LPS: lipopolysaccharide
RT-qPCR: quantitative reverse transcription PCR
Act D: actinomycin D
SIRT1: sirtuin 1
MeRIP qPCR: methylated RNA immunoprecipitation qPCR
STAT3: signal transducer and activator of transcription 3
COX2: cytochrome c oxidase subunit II
iNOS: inducible nitric oxide synthase
MHC-II: major histocompatibility complex II
FBS: fetal bovine serum
TBST: tris-buffered saline plus Tween-20
PBS: phosphate-buffered saline
ALKBH5: alkB homolog 5
EAU: experimental autoimmune uveitis
TNF- α: tumor necrosis factor- α
COP1: E3 ubiquitin ligase
USP18: ubiquitin specific peptidase 18
EP4: prostaglandin E receptor 4
IRF8: interferon regulatory factor 8
EGR1: early growth response 1
NF- κ B: nuclear factor κ B
EAE: experimental autoimmune encephalomyelitis.
