# Supplementary material for: Low Expression of YTH Domain-Containing 1 Promotes Microglial M1 Polarization by Reducing the Stability of Sirtuin 1 mRNA
Source: Front Cell Neurosci. 2021 Dec 15;15:774305. doi: 10.3389/fncel.2021.774305 (PMC8714917; doi:10.3389/fncel.2021.774305)
Supplement: Supplementary file 1 [file Data_Sheet_1.docx]

1.The original figures for WB





Figure2 C

The full scans of the entire original gels of YTHDC1





Figure2 C

The full scans of the entire original gels of ALKBH5





Figure2 C

The full scans of the entire original gels of GAPDH





Figure3 B

The full scans of the entire original gels of YTHDC1





Figure3 B

The full scans of the entire original gels of GAPDH





Figure3 D

The full scans of the entire original gels of iNOS





Figure3 D

The full scans of the entire original gels of GAPDH





Figure3 D

The full scans of the entire original gels of TNF-α





Figure3 D

The full scans of the entire original gels of GAPDH





Figure3 D

The full scans of the entire original gels of COX2





Figure3 D

The full scans of the entire original gels of GAPDH





Figure4 B

The full scans of the entire original gels of SIRT1





Figure4 B

The full scans of the entire original gels of USP18





Figure4 B

The full scans of the entire original gels of COP1





Figure4 B

The full scans of the entire original gels of EP4





Figure4 B

The full scans of the entire original gels of GAPDH





Figure4 D

The full scans of the entire original gels of SIRT1





Figure4 D

The full scans of the entire original gels of GAPDH





Figure4 F

The full scans of the entire original gels of SIRT1





Figure4 F

The full scans of the entire original gels of iNOS





Figure4 F

The full scans of the entire original gels of COX2





Figure4 F

The full scans of the entire original gels of GAPDH





Figure5 B

The full scans of the entire original gels of IRF8





Figure5 B

The full scans of the entire original gels of STAT3





Figure5 B

The full scans of the entire original gels of c/EBPβ





Figure5 B

The full scans of the entire original gels of EGR1





Figure5 B

The full scans of the entire original gels of GAPDH





Figure5 D

The full scans of the entire original gels of p-STAT3





Figure5 D

The full scans of the entire original gels of GAPDH





Figure5 F

The full scans of the entire original gels of STAT3





Figure5 F

The full scans of the entire original gels of p-STAT3





Figure5 F

The full scans of the entire original gels of GAPDH





Figure5 H

The full scans of the entire original gels of ac-STAT3





Figure5 H

The full scans of the entire original gels of GAPDH





Supplementary Figuer1 A

The full scans of the entire original gels of iNOS





Supplementary Figuer1 A

The full scans of the entire original gels of COX2





Supplementary Figuer1 A

The full scans of the entire original gels of TNFα





Supplementary Figuer1 A

The full scans of the entire original gels of GAPDH

2.The original figures for immunofluorescence

Figure3 F


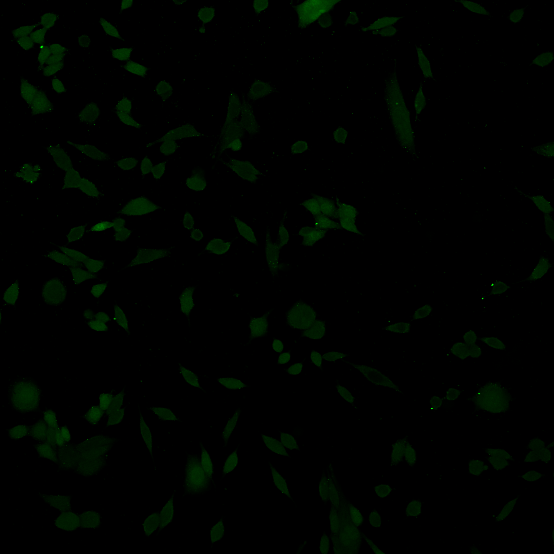

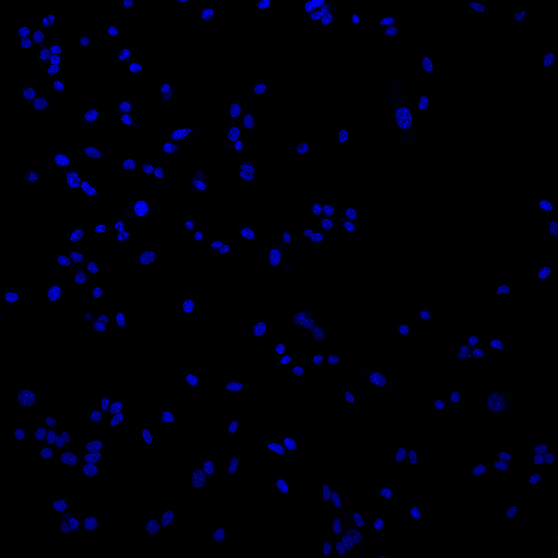


NC-iNOS NC-DAPI


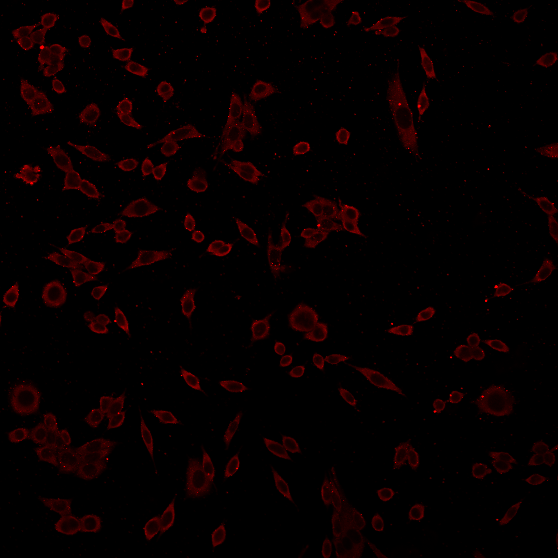

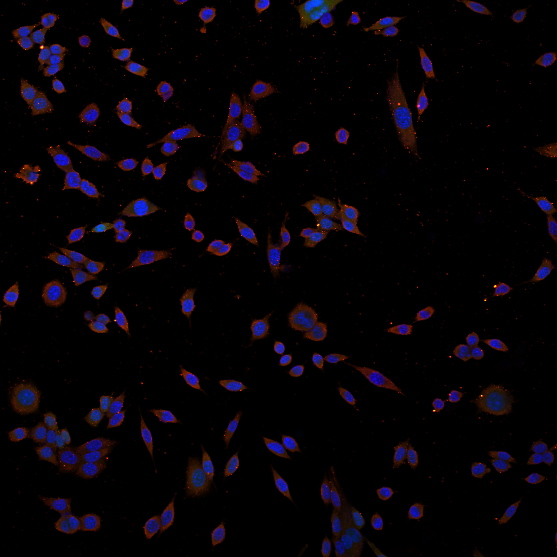


NC-IBA1 NC-Merge


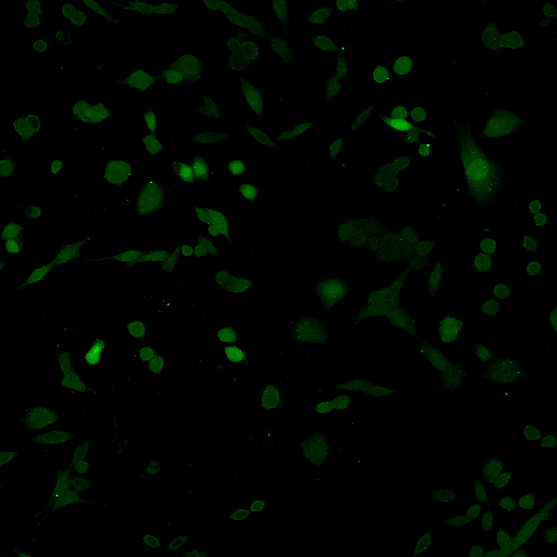

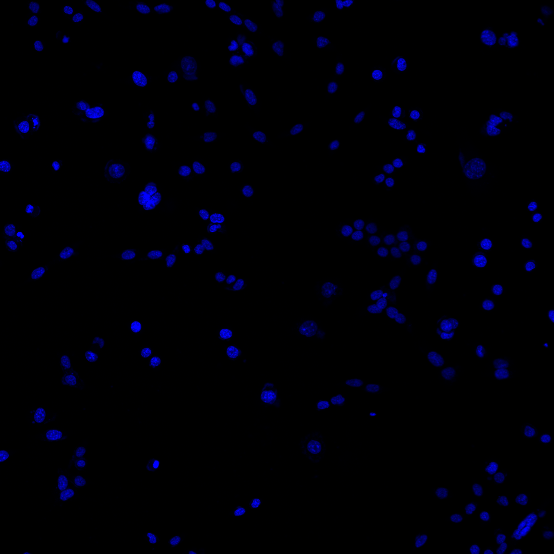


NC+LPS-iNOS NC+LPS-DAPI


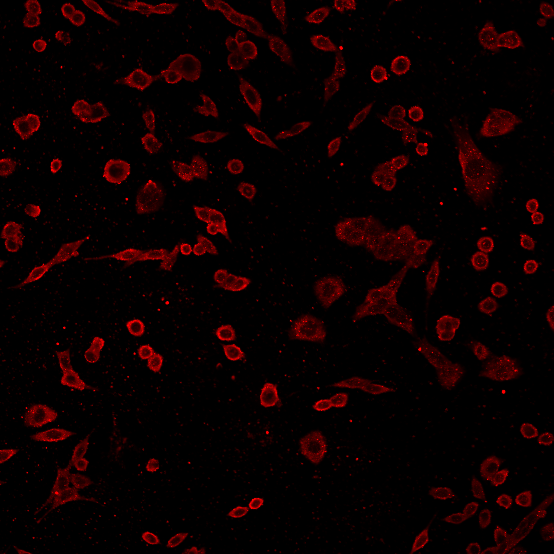

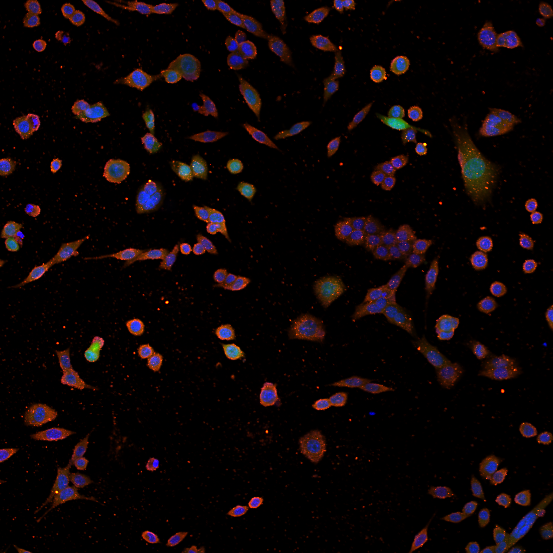


NC+LPS- IBA1 NC+LPS- Merge


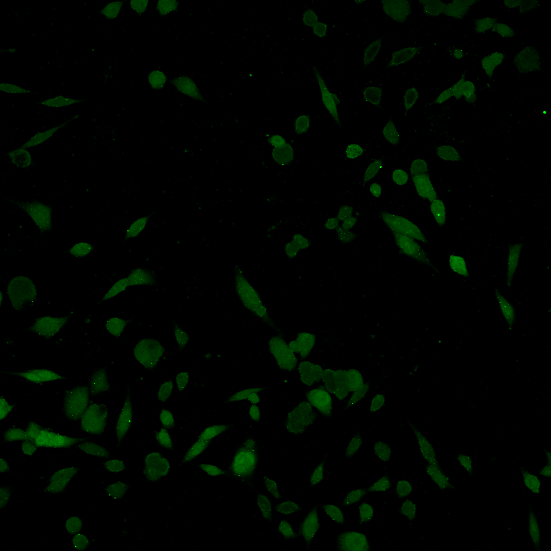

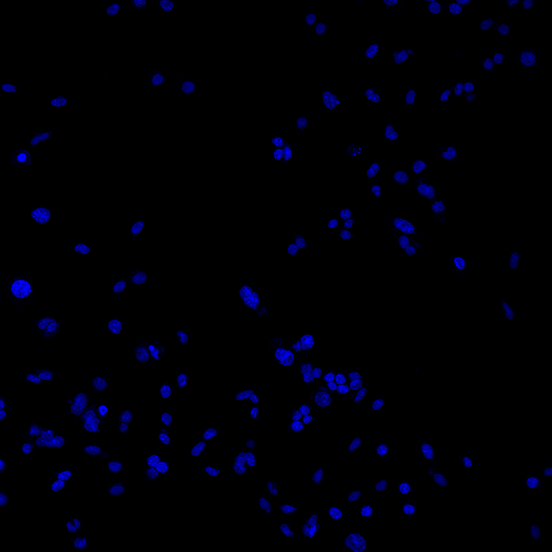


ShYTHDC1-iNOS ShYTHDC1-DAPI


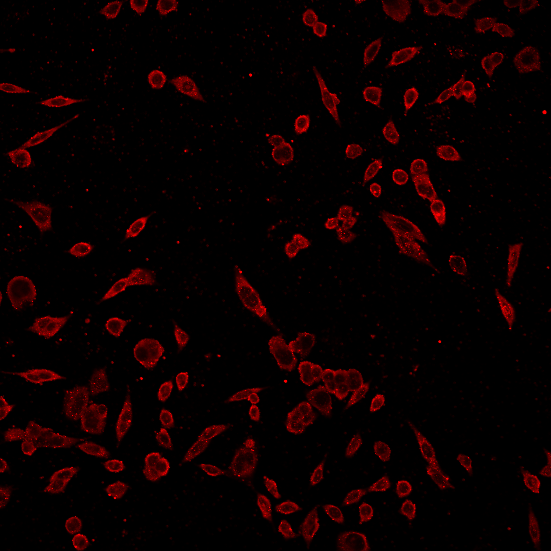

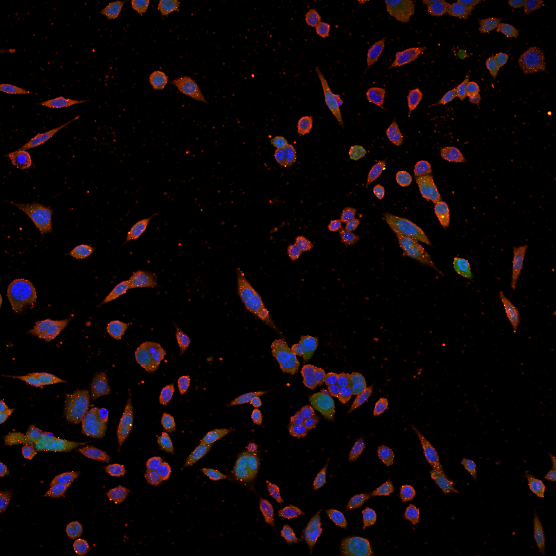


ShYTHDC1-IBA1 ShYTHDC1-Merge


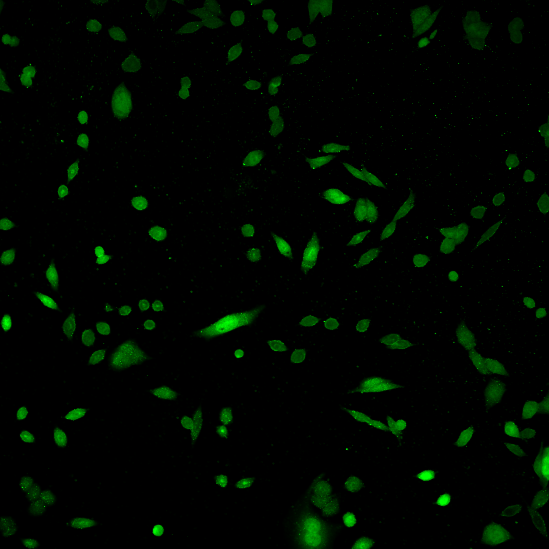

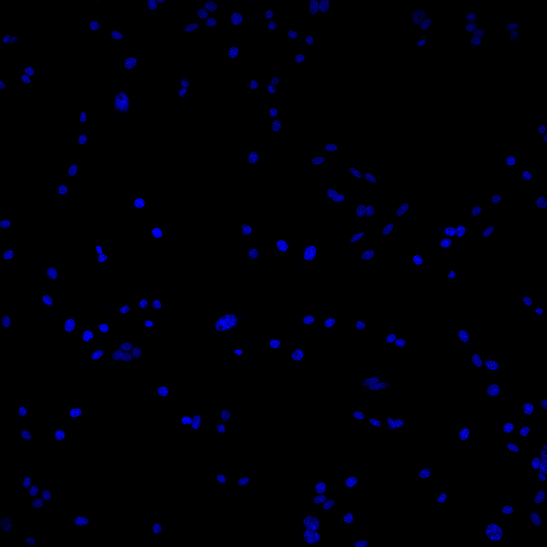


ShYTHDC1+LPS-iNOS ShYTHDC1+LPS-DAPI


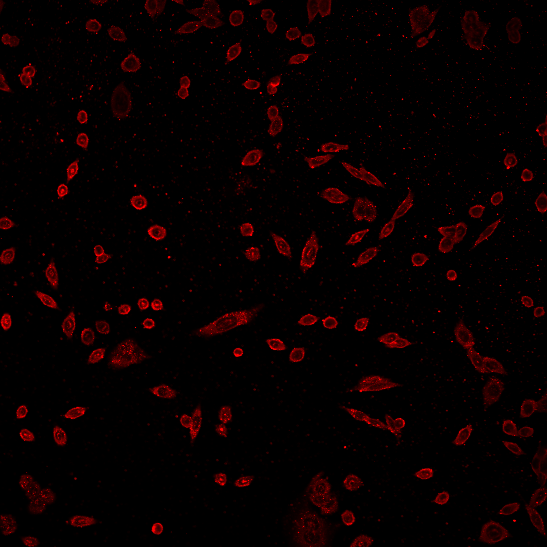

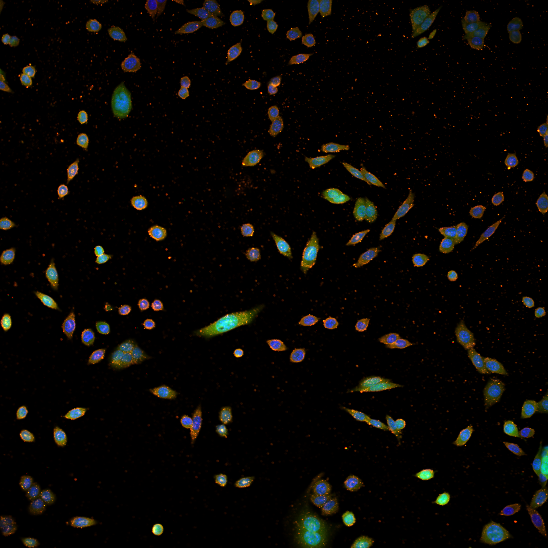


ShYTHDC1+LPS- IBA1 ShYTHDC1+LPS- Merge

Supplementary Figuer1 C


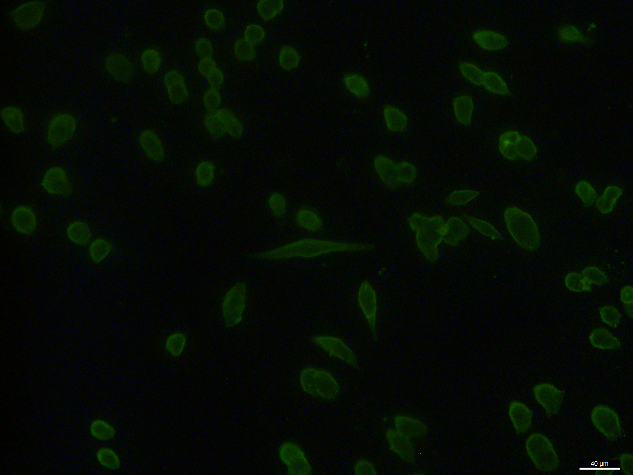

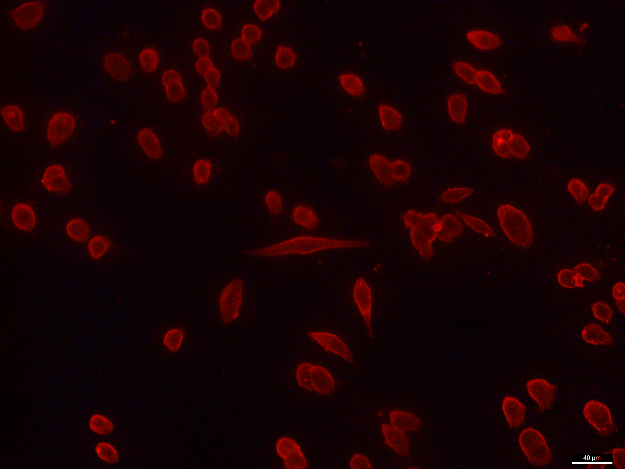


Control-iNOS Control-IBA1


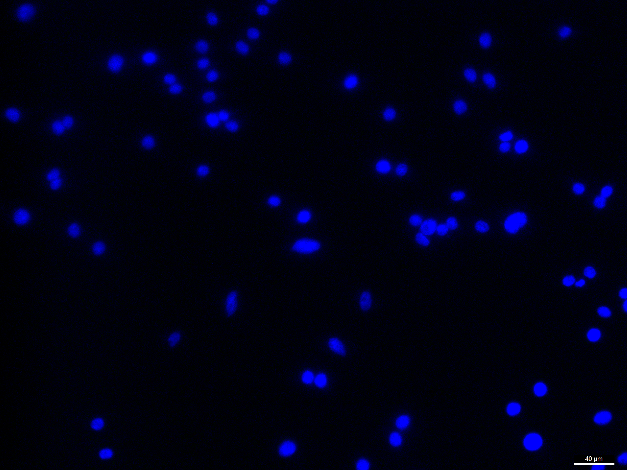

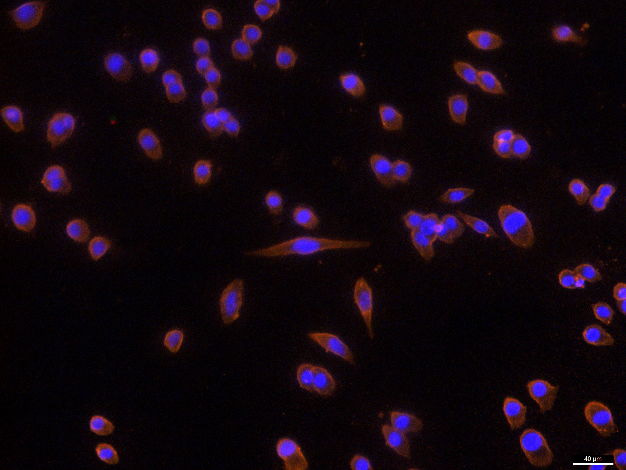


Control-DAPI Control-Merge


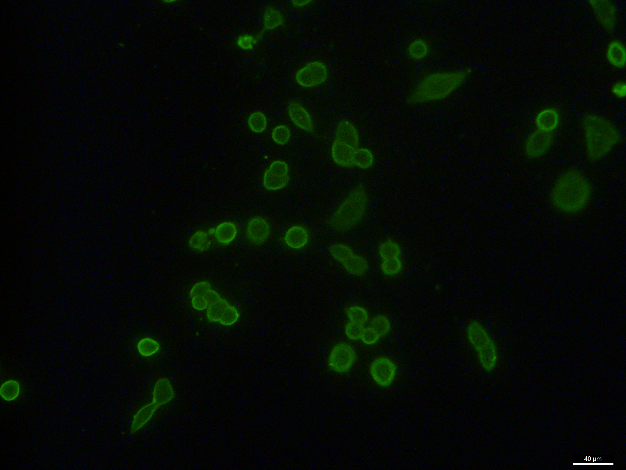

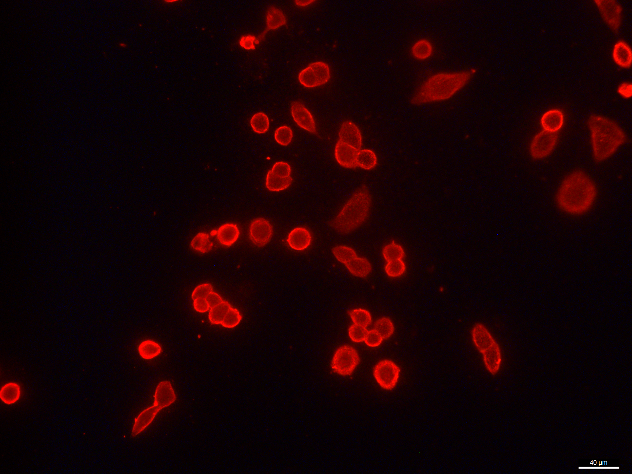


LPS-iNOS LPS-IBA1


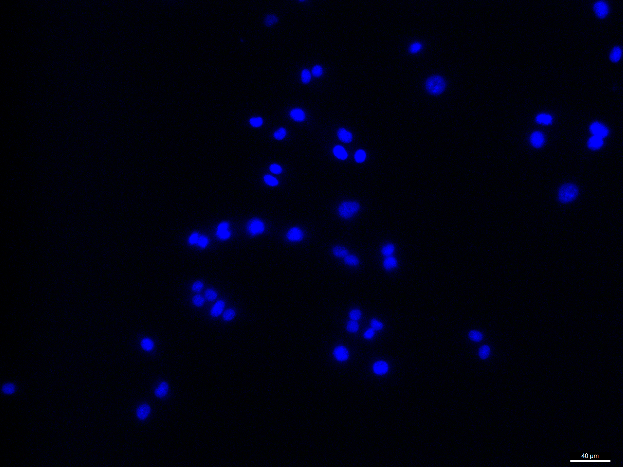

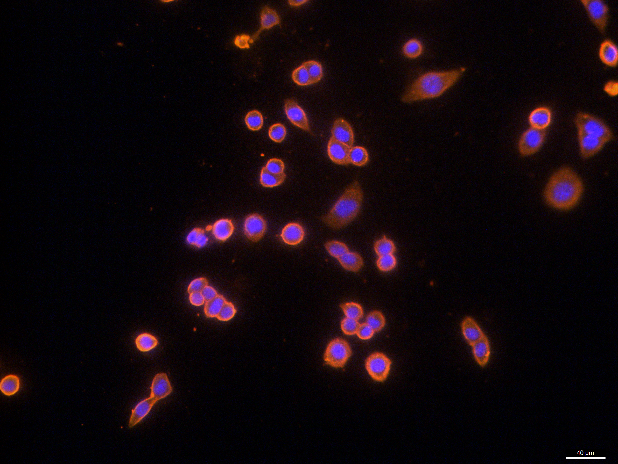


LPS-DAPI LPS-Merge

Supplementary Figuer1 H


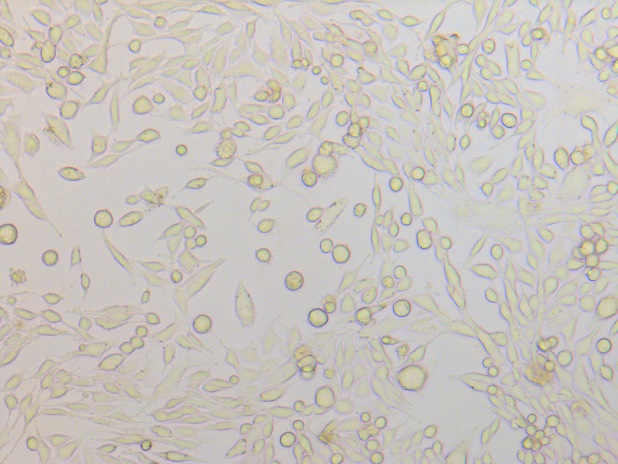

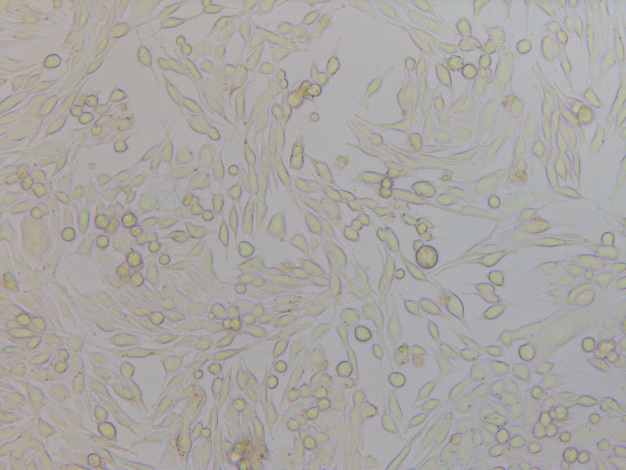


NC-Trans ShYTHDC1-1-Trans


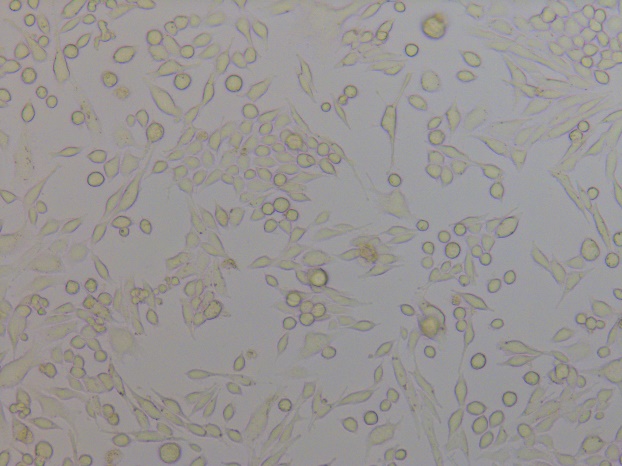

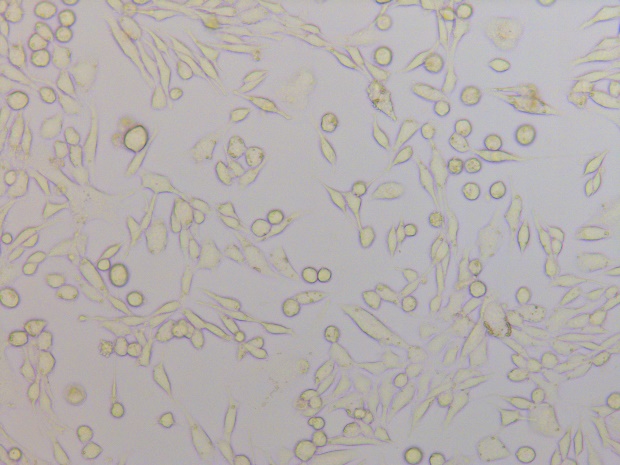


ShYTHDC1-2-Trans ShYTHDC1-3-Trans


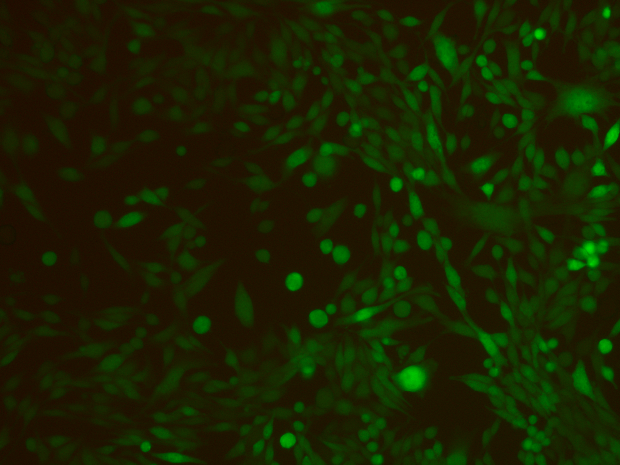

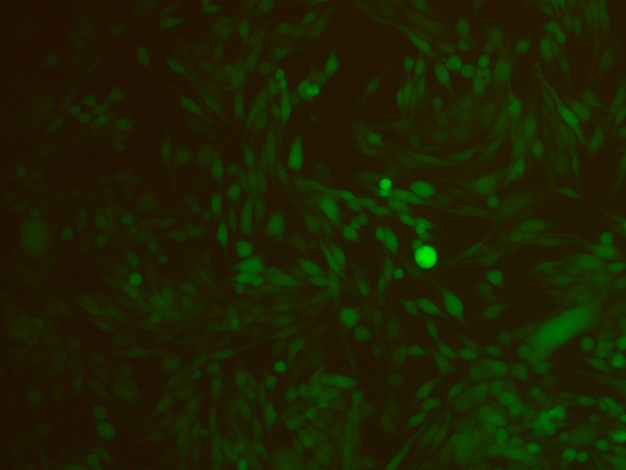


NC-GFP ShYTHDC1-1-GFP


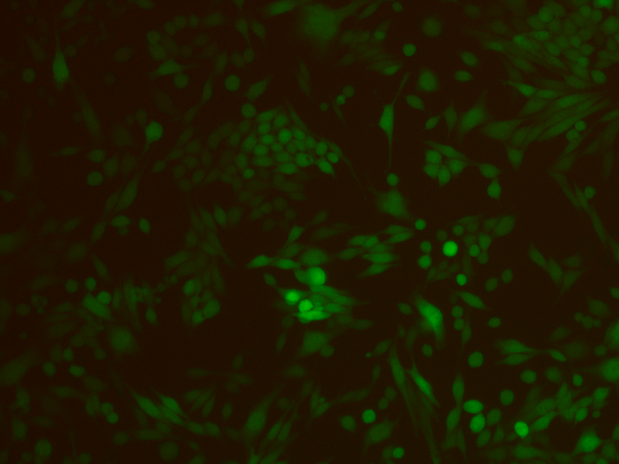

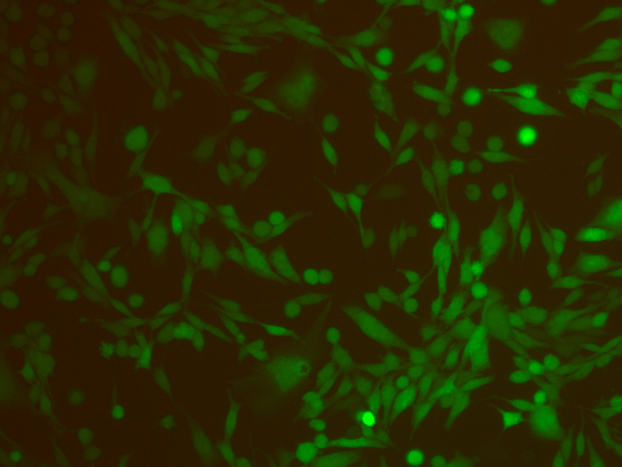


ShYTHDC1-2-GFP ShYTHDC1-3-GFP

3.The original figures for Tanswell

Figuer3 G


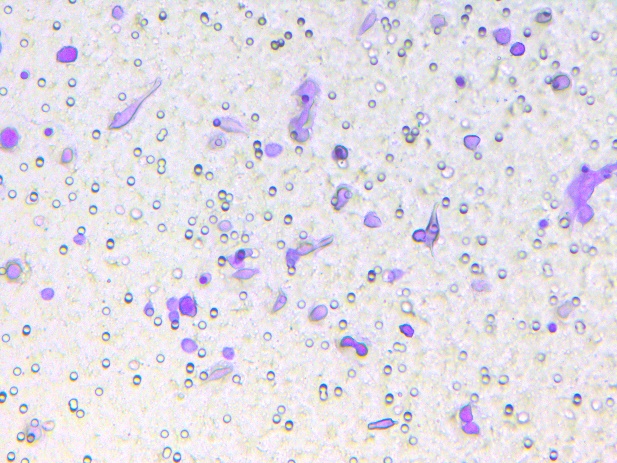

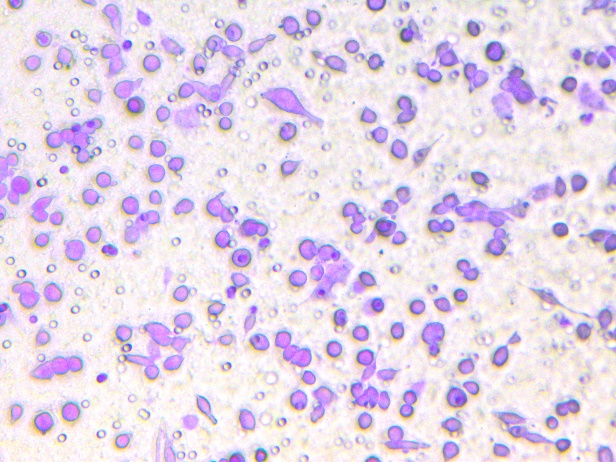


NC-Control NC-LPS


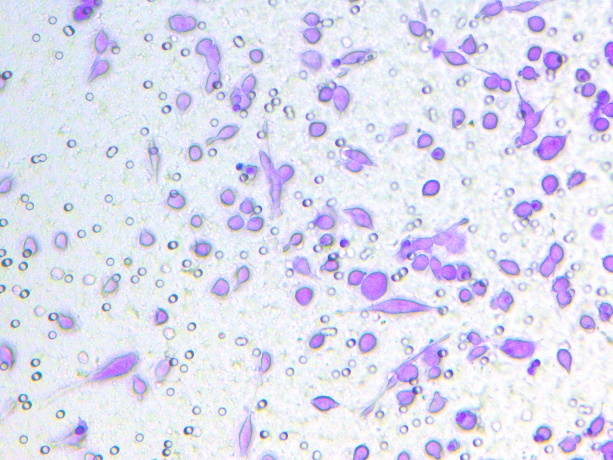

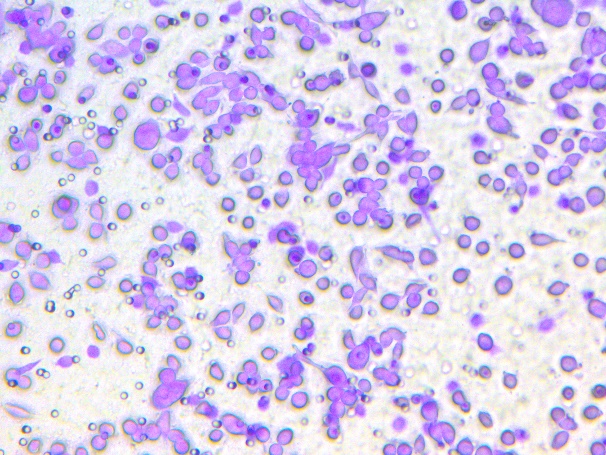


ShYTHDC1-Control ShYTHDC1-LPS

Supplementary Figuer1 F


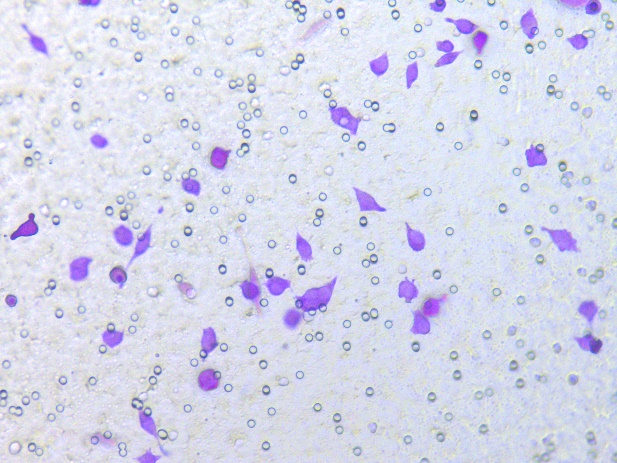

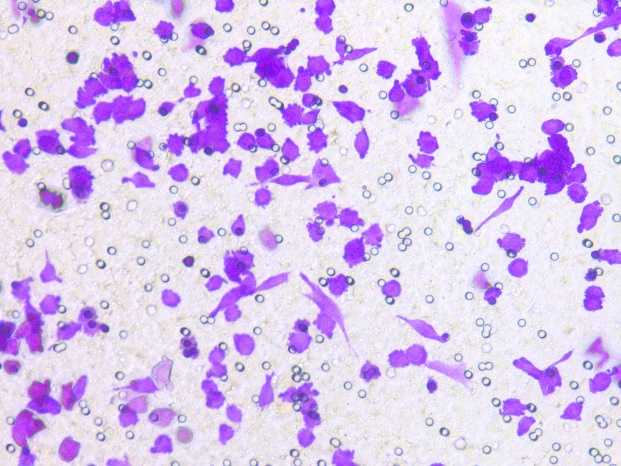


Control LPS

4.The original figures for wound heal

Figure3 I


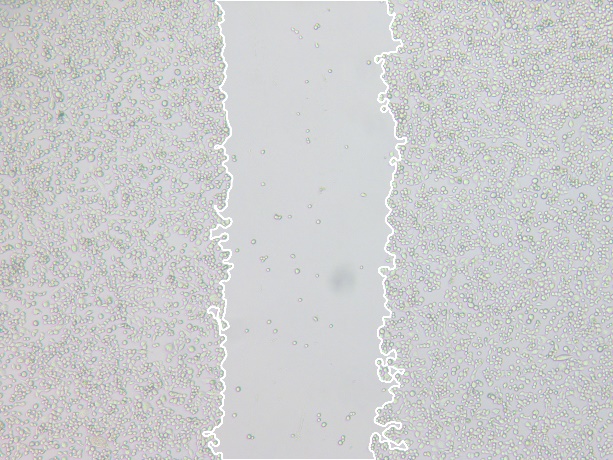

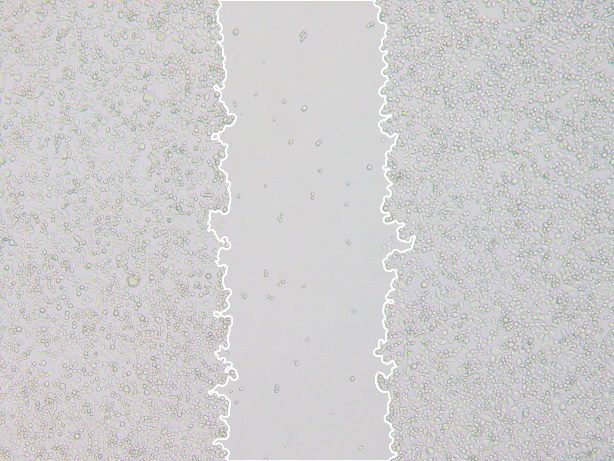


NC-Control-0h NC-LPS-0h


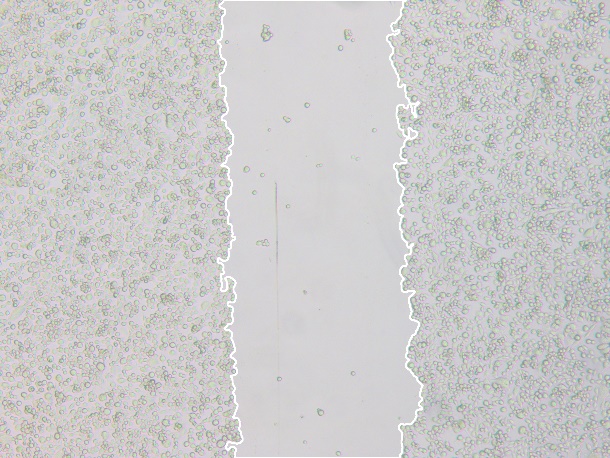

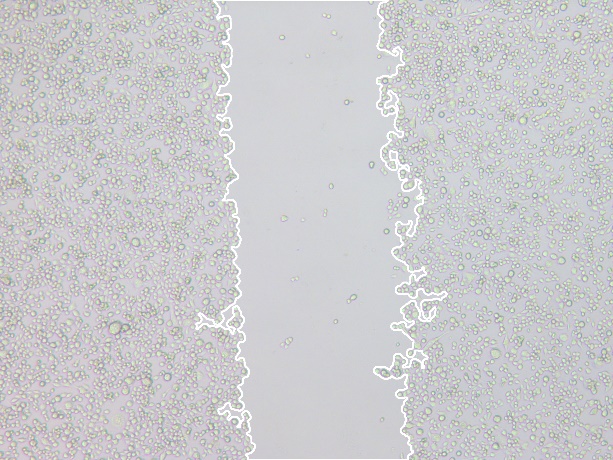


ShYTHDC1-Control-0h ShYTHDC1-LPS-0h


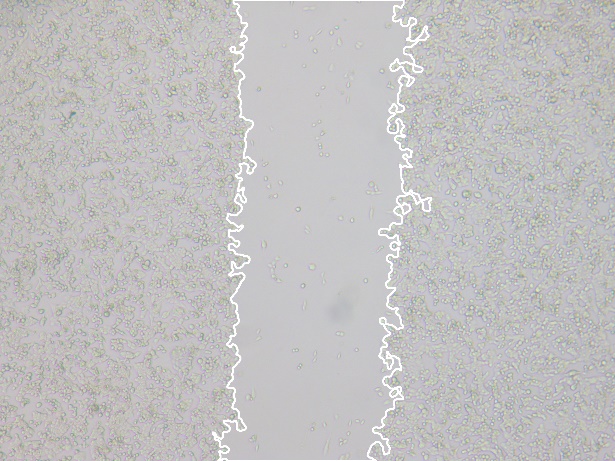

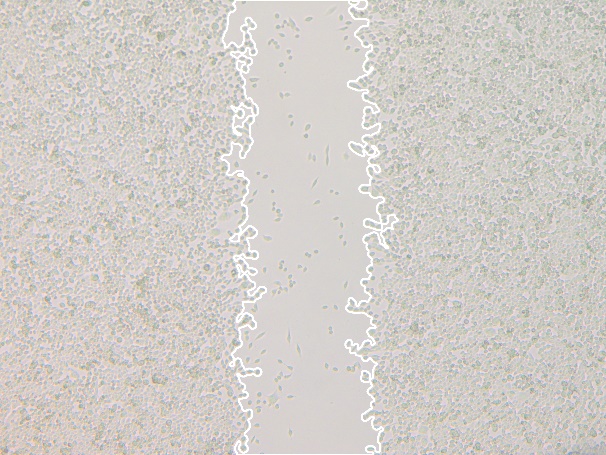


NC-Control-24h NC-LPS-24h


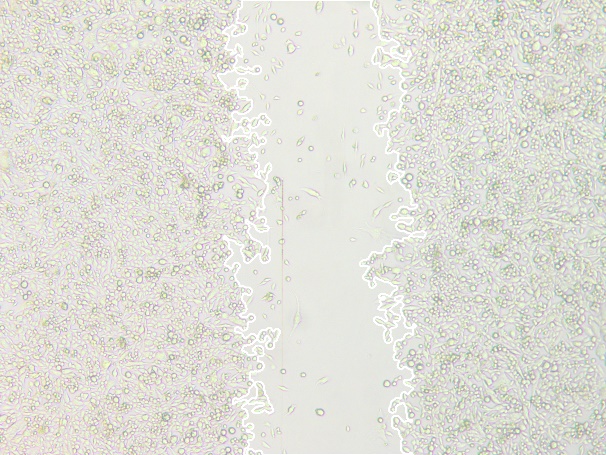

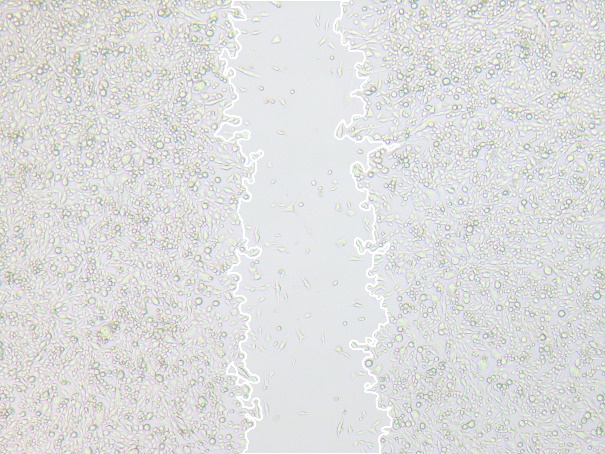


ShYTHDC1-Control-24h ShYTHDC1-LPS-24h

Supplementary Figuer1 F


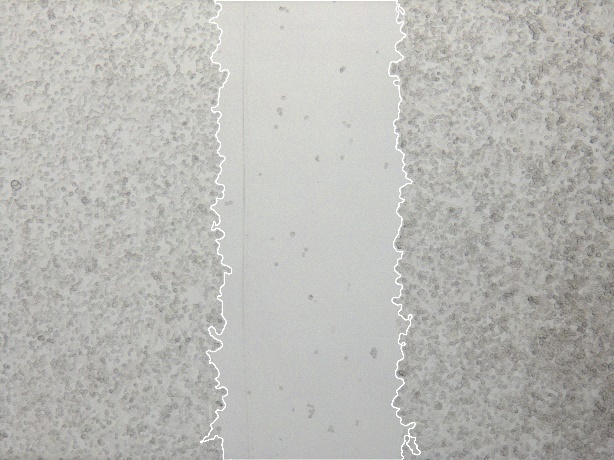

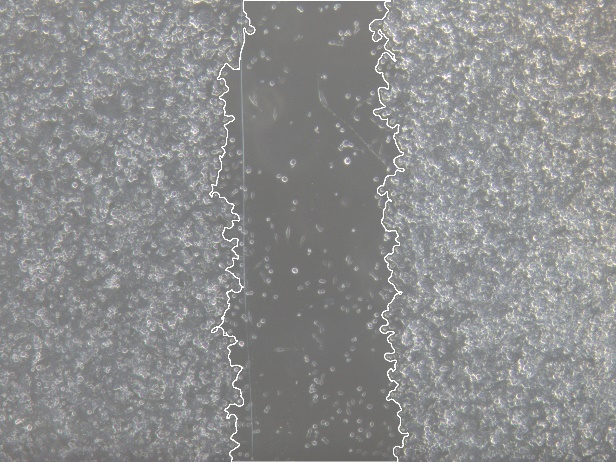


Control 0h Control 24h


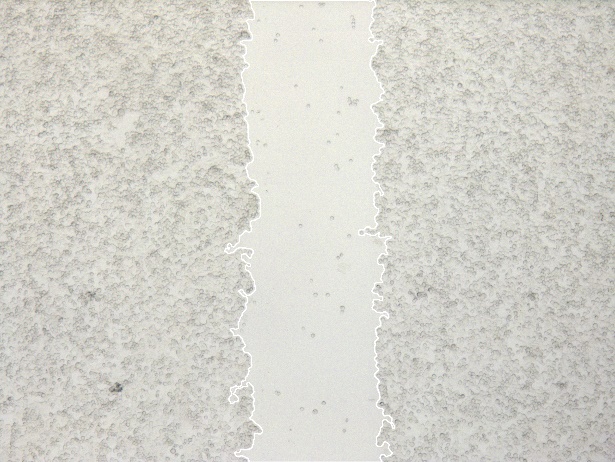

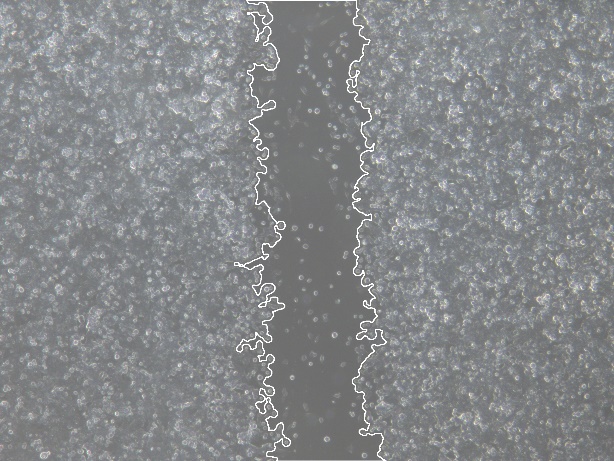


LPS 0h LPS 24h
