# Supplementary material for: Low Expression of YTH Domain-Containing 1 Promotes Microglial M1 Polarization by Reducing the Stability of Sirtuin 1 mRNA
Source: Front Cell Neurosci. 2021 Dec 15;15:774305. doi: 10.3389/fncel.2021.774305 (PMC8714917; doi:10.3389/fncel.2021.774305)
Supplement: Supplementary file 3 [file Data_Sheet_3.docx]

**Supplementary Figure 1.** (**A)**, (**B** )Western blot analysis of iNOS, COX2, and TNF-α in LPS (1 μg/ml)-induced BV2 cells at 24 h compared with the control. (**C)** Immunofluorescence analysis of iNOS in LPS (1 μg/ml)-induced BV2 cells 24 h compared with the control. Scale bar, 40 μm. **(D)**, **(E)** Wound healing analysis in BV2 cells induced by LPS compared with the control. Scale bar, 200 μm. (**F)**, **(G)** Transwell assay of LPS (1 μg/ml)-induced BV2 cells compared with the control. Scale bar, 200 μm. (**H)** Fluorescence microscopy image (GFP) and white light image (Trans) of BV2 cells transfected with lentivirus. NC: cells transfected with negative control shRNA; ShYTHDC1 (1, 2, 3): cells transfected with YTHDC1 shRNA. *P＜0.05, **P＜0.01, and *** P＜0.001 by Student´s t-test. The data are presented as the mean ± SD. (n=3).

**Table S1.** Primers for qRT-PCR.

| Gene | Forward Primer (5′-3′) | Reverse Primer (5′-3′) |
| --- | --- | --- |
| β-actin | CGTTGACATCCGTAAAGACCTC | CCACCGATCCACACAGAGTAC |
| YTHDC1 | GCTCATCTCACCAATCCCT | TGTCCATCACGTCCAATCT |
| COP1 | ACATGCAGCTCAGCTACAGAT | AGAGTAGAGGCCACTCATTTCTT |
| USP18 | TTGGGCTCCTGAGGAAACC | CGATGTTGTGTAAACCAACCAGA |
| IRAK1 | TCCTCCACCAAGCAGTCAAG | AAAACCACCCTCTCCAATCCT |
| EP4 | CCAAGAGGTGAGTGCTTCCC | CTGTTGTTCAGACTCTCTCCCT |
| SOCS1 | CTGCGGCTTCTATTGGGGAC | AAAAGGCAGTCGAAGGTCTCG |
| SIRT1 | GCTGACGACTTCGACGACG | TCGGTCAACAGGAGGTTGTCT |
| IKK | GGAGCCTGGGAAATGAAAGAA | GCCAGAGCCCTACCTGATTG |
| NFκB | GGAGGCATGTTCGGTAGTGG | CCCTGCGTTGGATTTCGTG |
| STAT1 | TCACAGTGGTTCGAGCTTCAG | GCAAACGAGACATCATAGGCA |
| IRF1 | ATGCCAATCACTCGAATGCG | TTGTATCGGCCTGTGTGAATG |
| IRF5 | GGTCAACGGGGAAAAGAAACT | CATCCACCCCTTCAGTGTACT |
| cEBPβ | CAAGAAGACGGTGGACAAGC | AGCTGCTCCACCTTCTTCTG |
| IRF8 | CGGGGCTGATCTGGGAAAAT | CACAGCGTAACCTCGTCTTC |
| RELA | AGGCTTCTGGGCCTTATGTG | TGCTTCTCTCGCCAGGAATAC |
| JUN | CCTTCTACGACGATGCCCTC | GGTTCAAGGTCATGCTCTGTTT |
| STAT3 | CAATACCATTGACCTGCCGAT | GAGCGACTCAAACTGCCCT |
| AR | CTGGGAAGGGTCTACCCAC | GGTGCTATGTTAGCGGCCTC |
| SP1 | GCCGCCTTTTCTCAGACTC | TTGGGTGACTCAATTCTGCTG |
| HIF1A | ACCTTCATCGGAAACTCCAAAG | ACTGTTAGGCTCAGGTGAACT |
| NFAT5 | ATCGCCCAAGTCCCTGTACT | GCTTGTCTGACTCATTGATGCTA |
| IRF3 | GAGAGCCGAACGAGGTTCAG | CTTCCAGGTTGACACGTCCG |
| TRAF6 | AAAGCGAGAGATTCTTTCCCTG | ACTGGGGACAATTCACTAGAGC |
| MAPK1 | GCCTTACTCTACTTCTCCCCA | CTGCCTCTGACTTCTGAATGC |
| SYK | CTACCTGCTACGCCAGAGC | GCCATTAAGTTCCCTCTCGATG |
| EGR1 | AGACGAGTTATCCCAGCCAAA | GGTCGGAGGATTGGTCATGC |

**Table S2.** Antibodies for Western blotting.

Primary

| Name | Source | Application and manufacturers |
| --- | --- | --- |
| Anti-GAPDH | Mouse Monoclonal | 1:5000, Proteintech, 60004-1-Ig |
| Anti-YTHDC1 | Rabbit monoclonal EPR21821 | 1:1000, Abcam, MA (ab1220159) |
| Anti-ALKBH5 | Rabbit monoclonal EPR18958 | 1:1000, Abcam, MA (ab195377) |
| Anti-TNF-α | Rabbit polyclonal | 1:500, Abcam, MA (ab6671) |
| Anti-iNOS | Rabbit monoclonal EPR16635 | 1:500, Abcam, MA (ab178945) |
| Anti-COX2 | Rabbit monoclonal | 1:1000, CST, #12282 |
| Anti-STAT3 | Rabbit monoclonal EPR787Y | 1:1000, Abcam, MA (ab68153) |
| Anti-IRF8 | Mouse monoclonal | 1:500, Santa Cruz, sc-365042 |
| Anti-pSTAT3 | Rabbit monoclonal EPR2147Y | 1:2000, Abcam, MA (ab176315) |
| Anti-c/EBPβ | Rabbit polyclonal | 1:1000, CST, #3087 |
| Anti-SIRT1 | Rabbit monoclonal | 1:1000, CST, #9475 |
| Anti-USP18 | Rabbit polyclonal | 1:1000, Immunoway, YT7591 |
| Anti-EGR1 | Mouse monoclonal | 1:100, Santa Cruz, sc-101033 |
| Anti-EP4 | Mouse monoclonal | 1:500, Santa Cruz, sc-55596 |
| Anti-COP1 | Rabbit polyclonal | 1:1000, Bethyl, A300-894A-T |
| Anti-acSTAT3 | Rabbit polyclonal | 1:500, Immunoway, YK0070 |

Secondary

| Name | Source | Application and manufacturers |
| --- | --- | --- |
| anti-Mouse IgG (H+L)-HRP | Goat polyclonal | 1:5000, Proteintech, SA00001-1 |
| anti-Rabbit IgG (H+L)-HRP | Goat polyclonal | 1:5000, Proteintech, SA00001-2 |

**Table S3.** Primers for MeRIP-qPCR.

| Gene | Forward Primer (5′-3′) | Reverse Primer (5′-3′) |
| --- | --- | --- |
| SIRT1-1 | GTTGCCACCAACACCTCTTC | GTTGCTTGGTCTACAAGTGTAGC |
| SIRT1-2 | AGCGATCGGCTACCGAGACA | GCGTGTGACGTTCTGTCATCGT |
