# Supplementary figures and images for: Low Expression of YTH Domain-Containing 1 Promotes Microglial M1 Polarization by Reducing the Stability of Sirtuin 1 mRNA
Source: Front Cell Neurosci. 2021 Dec 15;15:774305. doi: 10.3389/fncel.2021.774305 (PMC8714917; doi:10.3389/fncel.2021.774305)

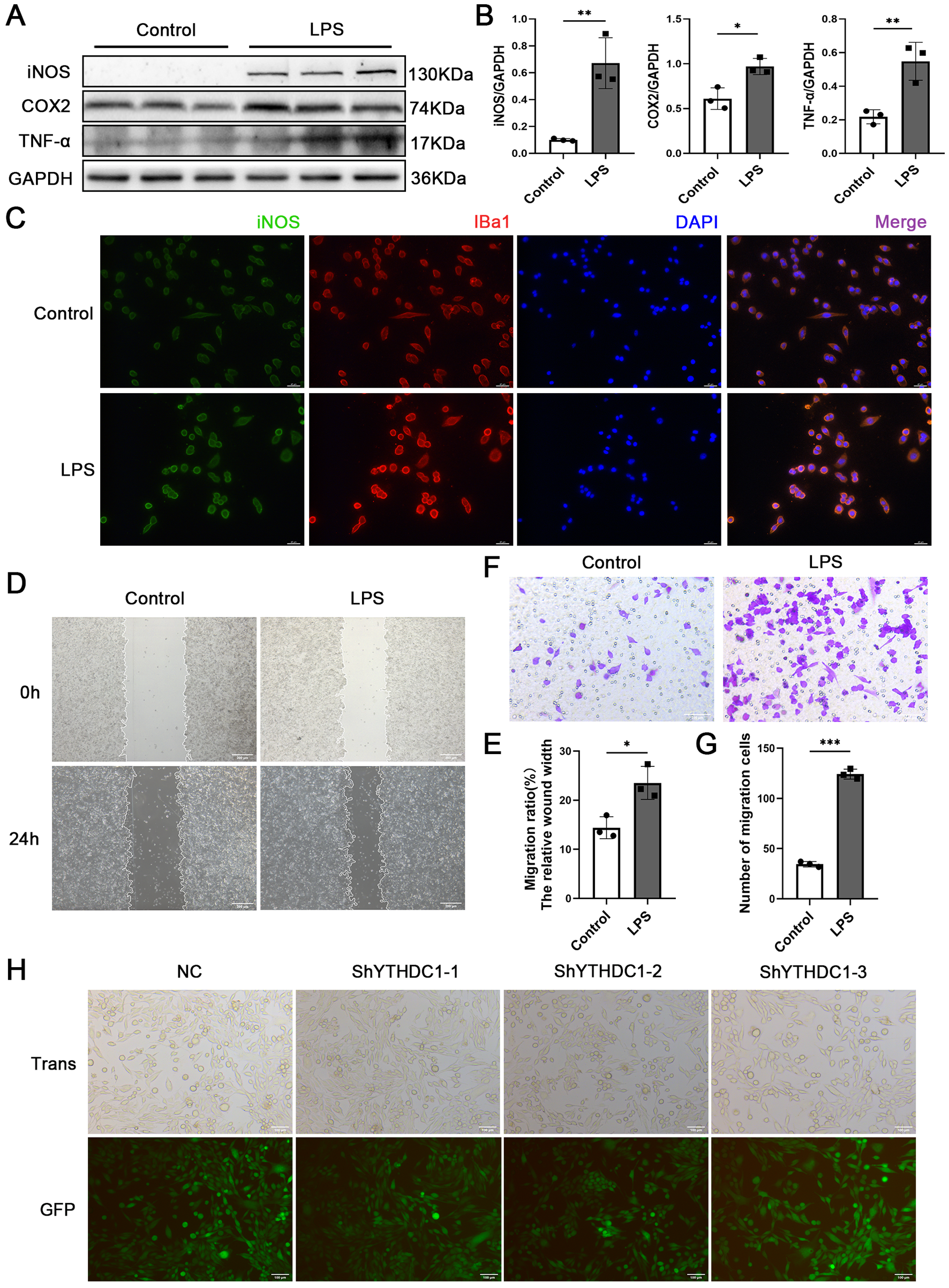

Supplement: Supplementary file 4 [file Image_1.TIF]
